# Posttraumatic stress symptomatology and abnormal neural responding during emotion regulation under cognitive demands: mediating effects of personality

**DOI:** 10.1017/pen.2020.10

**Published:** 2020-07-30

**Authors:** Michael Sun, Craig A. Marquardt, Seth G. Disner, Philip C. Burton, Nicholas D. Davenport, Shmuel Lissek, Scott R. Sponheim

**Affiliations:** 1 Department of Psychological and Brain Sciences, Dartmouth College, Hanover, NH, USA; 2 Department of Psychiatry and Behavioral Sciences, University of Minnesota, Minneapolis, MN, USA; 3 Minneapolis Veterans Affairs Health Care System, Minneapolis, MN, USA; 4 Department of Psychology, University of Minnesota, Minneapolis, MN, USA

**Keywords:** Posttraumatic stress, Attention, Emotion regulation, MMPI-2-RF, Veterans, fMRI

## Abstract

Posttraumatic stress disorder (PTSD) is often complicated by the after-effects of mild traumatic brain injury (mTBI). The mixture of brain conditions results in abnormal affective and cognitive functioning, as well as maladaptive behavior. To better understand how brain activity explains cognitive and emotional processes in these conditions, we used an emotional N-back task and functional magnetic resonance imaging (fMRI) to study neural responses in US military veterans after deployments to Iraq and Afghanistan. Additionally, we sought to examine whether hierarchical dimensional models of maladaptive personality could account for the relationship between combat-related brain conditions and fMRI responses under cognitive and affective challenge. FMRI data, measures of PTSD symptomatology (PTSS), blast-induced mTBI (bmTBI) severity, and maladaptive personality (MMPI-2-RF) were gathered from 93 veterans. Brain regions central to emotion regulation were selected for analysis, and consisted of bilateral amygdala, bilateral dorsolateral prefrontal (dlPFC), and ventromedial prefrontal/subgenual anterior cingulate (vmPFC-sgACC). Cognitive load increased activity in dlPFC and reduced activity in emotional responding brain regions. However, individuals with greater PTSS showed blunted deactivations in bilateral amygdala and vmPFC-sgACC, and weaker responses in right dlPFC. Additionally, we found that elevated emotional/internalizing dysfunction (EID), specifically low positive emotionality (RC2), accounted for PTSS-related changes in bilateral amygdala under increased cognitive load. Findings suggest that PTSS might result in amygdala and vmPFC-sgACC activity resistant to moderation by cognitive demands, reflecting emotion dysregulation despite a need to marshal cognitive resources. Anhedonia may be an important target for interventions that improve the affective and cognitive functioning of individuals with PTSD.

Psychologically traumatic events can have complex long-term effects on mental and physical health as well as impair personal and occupational functioning (Disner et al., 2017; Hoge et al., 2007; Pizarro et al., 2006). Military populations, in particular, experience a variety of life-threatening events that put them at an elevated risk for stress-related psychopathology. Returning US military personnel from Operations Enduring Freedom, Iraqi Freedom, and New Dawn endorsed high rates of exposure to explosive blasts in addition to posttraumatic stress disorder symptomatology (PTSS; Hoge et al., 2004, 2008; Schell & Marshall, 2008). Consequently, mild traumatic brain injury (mTBI) and posttraumatic stress disorder (PTSD) have been described as signature injuries of these conflicts (Hoge et al., 2008; Sayer, 2012; Stein et al., 2015; Warden, 2006). A diagnosis of PTSD is often confounded by mTBI, which complicates attempts to isolate clinical complaints to either PTSS or the after-effects of physical injuries, particularly those sustained during traumatic explosive blast events (Harvey & Bryant, 1998). In line with the theme of this special issue, we present findings identified through the lens of dimensional personality assessment to better understand the separable effects of PTSS and blast-induced mTBI (bmTBI) on neurophysiological responses among military veterans. We aimed to investigate candidate brain mechanisms that may explain co-occurring cognitive and emotional dysregulation specific to PTSS, taking into account the effects of bmTBI. Understanding the neurobiological underpinnings of maladaptive behavior after trauma could advance interventions that promote restoration of personal and occupational functioning and facilitate reintegration into civilian society.

Examining dimensions of symptomatology and personality in traumatized populations allows for a differentiation of how various aspects of self-reported experience map onto abnormal brain responses. Emerging research focusing on “subthreshold” PTSD (i.e., symptoms not meeting a full-threshold clinical diagnosis) has provided important insights into affective dysregulation, behavioral dysfunction, and suicide risk observed in traumatized populations (Cukor et al., 2010; Jakupcak et al., 2007, 2011; Marshall et al., 2001; Zlotnick et al., 2002). It is therefore justified to conceptualize the psychological after-effects of trauma as existing on a spectrum of severity without clear cut-offs between sick and well (Forbes et al., 2005; Ruscio et al., 2002). Statistical modeling of a dimension of PTSS may better reflect the range of possible maladaptive responses to trauma, and enhance statistical power to detect associations with other related aspects of psychopathology (Grove, 1991). Furthermore, dimensional models of PTSS appear capable of uncovering the associations with neurobiological systems sometimes not observable when using categorical diagnoses alone (Disner, Marquardt, Mueller, Burton, & Sponheim, 2018; Lieberman, Gorka, Funkhouser, Shankman, & Phan, 2017; Marquardt et al., 2018; Moran, 2016).

In this investigation, we aimed to more precisely characterize neural abnormalities associated with PTSS and blast exposure by using scales from the Minnesota Multiphasic Personality Inventory-2–Restructured Form (MMPI-2-RF; Tellegen & Ben-Porath, 2011). Two sets of indices composed of similar items – the Personality Psychopathology Five–Restructured Form (PSY-5-RF; Harkness et al., 2014) and the Higher Order scales (H-O; Sellbom, Ben-Porath, & Bagby, 2008; Tellegen & Ben-Porath, 2011) invoke personality frameworks from different traditions (to aid readability, see Table 1 for a list of acronyms). PSY-5-RF uses a structure similar to normative personality instruments developed using exploratory dimensionality reduction techniques (Harkness, McNulty, & Ben-Porath, 1995). By organizing items into five independent groupings, the scales resemble a clinical version of the commonly applied “Big Five” factor model of personality (e.g., Costa & McCrae, 1985). These include internalizing traits such as negative emotionality/neuroticism (NEGE-r) and introversion/low positive emotionality (INTR-r); externalizing traits such as aggressiveness (AGGR-r) and disconstraint (DISC-r); and disorganized thought processes reflective of psychoticism (PSYC-r). In contrast, the H-O scales were derived through an examination of the higher-order structure of the constituent Restructured Clinical (RC) scales within MMPI-2-RF (Ben-Porath, 2012; Sellbom et al., 2008). Self-report on these items is organized into three core groupings: emotional/internalizing dysfunction (EID), behavioral dysfunction (BXD), and thought dysfunction (THD). A three-factor H-O structure resembles the higher-order three-factor structure of psychiatric diagnoses (Kotov et al., 2011), and presents a more broad characterization of current functioning compared to PSY-5-RF scales. At the same time, the H-O scales can be further expanded into constituent RC scales for more detailed characterizations.

**Table 1.** Acronyms and their definitions

Acronym	Definition
DSM-IV-TR	Diagnostic and Statistical Manual IV – Text Revision
PTSD	Posttraumatic stress disorder
PTSS	Posttraumatic stress symptoms
mTBI	Mild traumatic brain injury
bmTBI	Blast-induced mild traumatic brain injury
fMRI	Functional Magnetic Resonance Imaging
ROI	Region of Interest
Brain areas	
dlPFC	Dorsolateral prefrontal cortex
vmPFC-sgACC	Ventromedial prefrontal cortex-subgenual anterior cingulate cortex
MMPI-2-RF	Minnesota multiphasic personality inventory 2 Restructured Form
Validity Metrics:	
L-r	Uncommon virtues
K-r	Adjustment validity
F-r	Infrequent Responses
Fp-r	Infrequency Psychopathology
TRIN-r	True Response Inconsistency
VRIN-r	Variable Response Inconsistency
PSY-5-RF	Personality Psychopathology-Five Scales Restructured Form
PSY-5-RF Scales:	
NEGE-r	Negative Emotionality/Neuroticism
INTR-r	Introversion/Low Positive Emotionality
AGGR-r	Aggressiveness
DISC-r	Disconstraint
PSYC-r	Psychoticism
H-O	Higher-Order Scales
H-O Scales:	
EID	Emotional/Internalizing Dysfunction
BXD	Behavioral Dysfunction
THD	Thought Dysfunction
RC	Restructured Clinical scales
RCd	Restructured Clinical Scale for Demoralization
RC2	Restructured Clinical Scale for Low Positive Emotions
RC7	Restructured Clinical Scale for Dysfunctional Negative Emotions
RC4	Restructured Clinical Scale for Antisocial Behavior

The PSY-5-RF and H-O scales provide complementary frameworks for understanding PTSS-related expressions of internalizing and externalizing dysfunction. Across various studies of personality, PTSD has been most strongly linked to high neuroticism, followed by low conscientiousness and low extraversion (Kotov, Gamez, Schmidt, & Watson, 2010). Variability in these or similar dimensions of personality may help explain differences across people in terms of their PTSS and psychiatric comorbidities (Miller, 2003; Miller, Greif, & Smith, 2003; Miller et al. 2012). Many individuals with posttraumatic stress commonly endorse elevated negative emotionality (Miller, Kaloupek, Dillon, & Keane, 2004). However, those with greater internalizing symptoms (e.g., mood disorders) also report lower positive emotionality/extraversion, while those with externalizing symptoms (e.g., disruptive substance use) report reduced constraint/conscientiousness. For example, NEGE-r was the primary PSY-5-RF index separating military veterans with PTSD from comparison controls in one post-deployment sample (Arbisi, Polusny, Erbes, Thuras, & Reddy, 2011). Furthermore, EID and all of its component RC subscales (RCd – demoralization; RC2 – low positive emotions; and RC7 – dysfunctional negative emotions), along with RC4 (antisocial behavior), distinguished PTSD from controls (Arbisi et al., 2011). In particular, RC7 (dysfunctional negative emotions), which includes items about intrusive thoughts, rumination, and nightmares, has been replicated as an important distinguishing index (Wolf et al., 2008). It may be that distress and generalized impairment commonly observed with bmTBI may be accounted for by emotional disruptions associated with comorbid elevations in PTSS. To our knowledge, no study to date has used the PSY-5-RF or H-O scales to explain PTSS-related cognitive and neurophysiological dysfunction using functional magnetic resonance imaging (fMRI).

Findings regarding the physiology of emotion regulation provide a neuroanatomical framework for understanding anxiety and traumatic stress-related cognitive impairments. Such perspectives (e.g., Etkin, Büchel, & Gross, 2015; Gross, 2015) posit that brain regions central to the generation of negative affect are modulated through pathways of model-based mechanisms of explicit regulation or model-free mechanisms of implicit regulation (Figure 1). The dorsolateral prefrontal cortex (dlPFC) may take an explicit role in regulating anxiety by changing a person’s model (i.e., their understanding) of threat (e.g., from “bad for me” to “good for me”; Corbetta & Shulman, 2002). Furthermore, a high cognitive load in various attentional control paradigms has been strongly associated with dlPFC activation (Curtis & D’Esposito, 2003; Tsuchida & Fellows, 2008). DlPFC may assist with compensatory processing during states of anxiety by actively inhibiting responses to distractors, shifting of attention, and updating working memory. Eysenck’s attentional control theory suggests that threat processing may become disruptive in certain circumstances when efforts to disengage using explicit attentional control depletes the same cognitive resources required for other relevant tasks (Eysenck, Derakshan, Santos, & Calvo, 2007). In line with this, individuals with PTSS commonly report hyperactive threat processing in daily life. Consequentially, they must use goal-directed attentional systems to manage the psychological impact of trauma-related cues from their external (e.g., unpleasant reminder images) and internal (e.g., personal worries, negative evaluations, memories of trauma) environments. These individuals may feel particularly compromised when other tasks in their lives require use of those same cognitive inhibition and shifting abilities (Berggren & Derakshan, 2013; Miyake et al., 2000). In other words, initial maladaptive processing of task-irrelevant threat and overburdened compensatory responses may explain some aspects of cognitive dysfunction among individuals with elevated PTSS.

**Figure 1.**
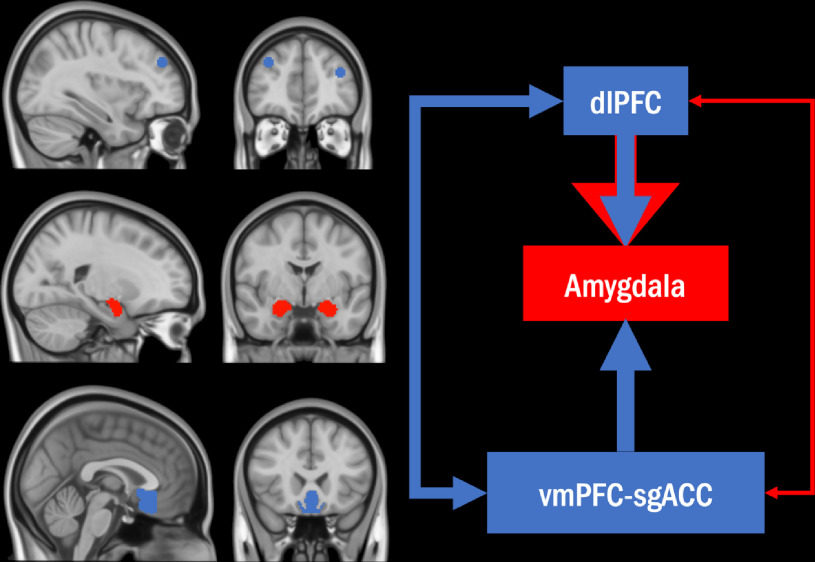
Neuroanatomical framework of emotional regulation with study regions of interest.

*Note:* On the leftmost column, left dlPFC depicted at *x* = −36, *y* = 44, *z* = 22 (top left); left amygdala depicted at *x* = −24, *y* = −3, *z* = −18; vmPFC-sgACC depicted at *x* = 0, *y* = 22, *z* = −14. A systems neuroscience model of explicit and implicit emotion regulation is displayed for the study regions of interest adapted from (Etkin et al., 2015). Red outlines and arrows suggest possible influences of anxiety in accordance with attentional control theory (Eysenck et al., 2007). vmPFC = ventromedial prefrontal cortex, sgACC = subgenual anterior cingulate cortex, dlPFC = dorsolateral prefrontal cortex.

For tasks that require little cognitive effort, explicit regulatory compensation may not be employed. However, anxiety-related deficits may still be observable via other neural mechanisms. For example, Fales and colleagues (2008) observed that greater anxious symptomatology was associated with broadly reduced neural activity at rest. Implicit regulatory regions such as vmPFC-sgACC have also been posited to regulate emotional responding in a model-free (i.e., experience-based) manner. These areas also play a role in generating and updating inhibitory responses through new perceptions of stimuli (e.g., Eysenck et al., 2007; Öhman & Mineka, 2001), and are implicated as updating abilities appear to be consistently disrupted in PTSD. These results also suggest a greater cognitive resource utilization with increased anxiety, making it conceivable that dlPFC-mediated compensatory activity may influence vmPFC-sgACC activity. The amygdala has a well-validated role in attentional capture from affective information (LeDoux, 2012; Öhman, Flykt, & Esteves, 2001; Pessoa & Ungerleider, 2004; Vuilleumier, 2005; Vuilleumier & Huang, 2009), and contributes to the expression of anxiety. It would be expected that higher levels of anxiety and presentation of threat cues would be associated with increased amygdala activation.

In the current study, we examined the associations for dimensional measures of PTSS and bmTBI with brain responses within an emotion regulation neural system composed of dlPFC, vmPFC-sgACC, and amygdala during an N-back task involving the manipulation of cognitive and affective load. We used a multiply-mediated moderation framework to account for brain activity with respect to personality dysfunction using the PSY-5-RF and H-O scales. The affective manipulation within the N-back task was anticipated to partially deplete goal-directed attentional resources necessary for disengagement from threatening images. In this compromised cognitive state, individuals with elevations in PTSS were expected to be inefficient at inhibiting their automatic processing of threatening images.

## 1. Methods

### 1.1. Participants

The initial sample (*n* = 115, M_age_ = 34.36, SD_age_ = 8.58) consisted of US military veterans (111 male, 96.52%) who completed the study protocol at the Minneapolis Veterans Affairs Health Care System and the University of Minnesota. All participants were previously deployed to combat zones as part of Operation Enduring Freedom or Operation Iraqi Freedom. Racial/ethnic self-identification of the participants consisted of 83.48% white, 3.48% black, 0.87% Asian American, 0.87% Native American, and 11.30% other. Participants were excluded if they shared evidence of current or past unstable medical conditions that would likely alter brain functioning (e.g., clear anoxic episode, current uncontrolled diabetes); neurological conditions; current DSM-IV-TR psychotic disorders; current or past DSM-IV-TR substance dependence other than alcohol, caffeine, or nicotine; current DSM-IV-TR substance abuse other than alcohol, caffeine, or nicotine; or current or past formal diagnosis of attention-deficit/hyperactivity disorder. Exclusion also occurred when participants reported head injury with a loss of consciousness >30 minutes, post-traumatic amnesia for >24 hours, skull fracture, positive neuroradiological findings, or hospitalization for >24 hours due to a head injury (i.e., TBI that was moderate in severity or greater). Frequent boxers and kickboxers were excluded. Participants who tested positive for elevated blood alcohol content on the day of study were excluded. In keeping with a cross-sectional design that evaluates the range of typical post-deployment functioning among veterans, individuals currently receiving mental health treatment were not asked to alter ongoing care. The institutional review boards of the Minneapolis Veterans Affairs Health Care System and the University of Minnesota approved the study. We assert that all procedures contributing to this work comply with the ethical standards of the relevant national and institutional committees on human experimentation and with the Helsinki Declaration of 1975, as revised in 2008.

### 1.2. Clinical assessment

*Clinician-Administered PTSD Scale for DSM-IV (CAPS), Fourth Edition.* CAPS is a clinician-administered semi-structured interview measure designed to assess PTSD symptomatology (Blake et al., 1995; Weathers, Keane, & Davidson, 2001). Symptoms are scored for frequency and intensity using a five-point scale (0–4). For this study, a general PTSD severity index was computed by summing all symptom frequency and intensity scores across the various PTSS domains.

*Minnesota Blast Exposure Screening Tool (MN-BEST).* MN-BEST is a semi-structured TBI screening instrument used to evaluate the severity of an individual’s three most significant concussive blast-related events (Nelson et al., 2011). Events were classified as blast-related if the participant reported feeling the pressure wave and attributed the after-effects to the blast, though secondary and tertiary injuries were common. Each self-reported possible bmTBI event was classified on the basis of acute-stage injury parameters outlined by the American Congress of Rehabilitation Medicine (Kay et al., 1993), including loss of consciousness no more than 30 minutes in duration and post-traumatic amnesia no more than 24 hours in duration. Reported bmTBIs were reviewed by clinical neuropsychologists and evaluated as to whether the injuries plausibly met the minimal biomechanical threshold of concussion (McCrea, 2007). Raters assigned composite bmTBI severity ratings to incidents based on a modified version of the scoring scheme proposed by Ruff and Richardson (1999). The maximum score for a single bmTBI event was 3, so the maximum score across the three possible events rated was 9.

*Minnesota Multiphasic Personality Inventory-2–Restructured Form (MMPI-2-RF).* Valid responding on MMPI-2-RF was determined through an examination of participant scores on the following scales: uncommon virtues (L-r), adjustment validity (K-r), infrequent responses (F-r), infrequency psychopathology (Fp-r), true response inconsistency (TRIN-r), and variable response inconsistency (VRIN-r) scales. Based on the criteria outlined by Ben-Porath (2012), participant profiles with F-r = 120, Fp-r ≥ 100, L-r ≥ 80, K-r ≥ 70, or with TRIN-r or VRIN-r ≥ 80 were excluded from analysis (*n* = 22).

## 2. Combat N-back task protocol

**Figure 2.**
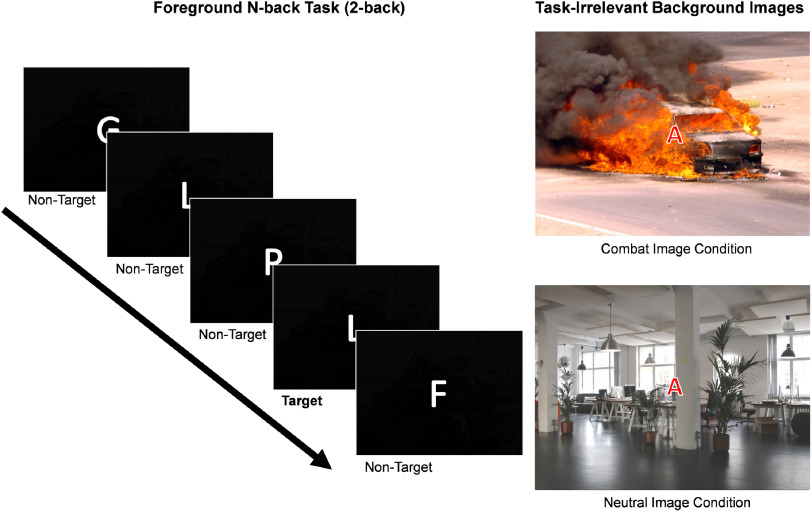
Affective N-back task design.

*Note:* The emotional N-back task design of the study. Example of trials from the 2-back condition (left). Examples of the combat and neutral affective background image conditions are displayed using substitute images from freely available online sources (right).

The N-back task stimuli consisted of single letters centered on a screen superimposed over task-irrelevant neutral or combat background images (Figure 2). Participants were tasked with identifying target letters during counter-balanced manipulations of cognitive load (0-back vs. 2-back) and affective content (neutral vs. combat images). During 0-back trials, participants pressed a response button when designated target letters appeared (e.g., Target = “A,” FAHRALPKAQ). For the 2-back condition, participants indicated when a sequentially presented letter was identical to the letter presented two screens before (e.g., GLPLFGNRNR). Low-arousal and intermediate-valence (i.e., neutral pleasantness) background images were selected using the International Affective Picture System (IAPS; Lang, Bradley, & Cuthbert, 2005) based on published ratings (IAPS identifiers 2383, 2393, 2880, 7050, 7080, 7175, 7205, 7224, 7550, 7705). Ten images of aversive Operation Iraqi Freedom–related combat scenes were also selected from a larger set of stimuli used in a previous study of post-deployment functioning (Marquardt et al., 2018). These images depicted scenes with threatening enemy combatants, civilian injuries, and roadside bombings. Participants previously rated these combat scenes as highly arousing and unpleasant.

N-back letters and background images were presented simultaneously for 1000 ms followed by inter-trial intervals of 1100 ms with centrally located crosshairs. Participants were allowed to respond until the onset of the next stimulus, but were asked to make their selections as quickly as possible while ignoring the background images. Task trials advanced regardless of the responses provided by participants to prevent a possible negative reinforcement for quicker button presses. Task trials were administered in blocks of 10 with 2–4 target trials per block and 32 total blocks administered across two separate runs. Overall, participants viewed eight total blocks from each combination of experimental manipulations, and experienced 25 0-back neutral, 26 2-back neutral, 25 0-back combat, and 24 2-back combat target trials interspersed with non-target trials in those same blocks. Non-target trials did not necessitate responses. Stimulus delivery was controlled by E-Prime software (Psychology Software Tools, 2012, Sharpsburg, PA).

### 2.1. Functional MRI

*Image acquisition.* All fMRI scans were conducted at the Center for Magnetic Resonance Research at the University of Minnesota on a Siemens Trio 3 T scanner (Siemens, Erlangen, Germany) with a 32-channel receive-only phased array radio frequency head coil. Anatomical scans with 1 mm isotropic resolution (224 coronal slices) were obtained using a T1-weighted MPRAGE sequence (TR = 2530 ms, TE = 3.65 ms, T1 = 1100 ms, flip angle = 7 degrees, FOV = 176 × 256 mm). Functional scans were gradient echo EPI images consisting of 348 volumes with 2 mm isotropic resolution, each with 64 slices (TR = 1320 ms, TE = 30 ms, flip angle = 90 degrees, multiband factor = 4, matrix = 106 × 106, FOV = 212 mm, 2 mm thick).

*Preprocessing.* Image analysis was performed using Analysis of Functional Neuroimages (AFNI) software (Cox, 1996). Each subject’s data were motion-corrected such that all subsequent volumes of both N-back runs were registered to the first volume of first N-back run. Data were smoothed with a Gaussian blur of 4.5 mm full-width-at-half-maximum using AFNI’s 3dBlurToFWHM. Distortion reduction was achieved using the top-up command from the FMRIB Software Library (FSL) and an EPI scan with identical parameters to those of the task, but with the opposite phase-encoding direction and thus the opposite pattern of distortion (Andersson et al., 2003; Smith et al., 2004). Within the framework of a general linear model, blood oxygen level-dependent (BOLD) responses were analyzed at the individual subject level to produce separate beta weights for each of the 0-back neutral, 0-back combat, 2-back neutral, and 2-back combat block conditions. These beta weights were treated as dependent variables in the subsequent multi-subject analyses. Six motion parameters, five degrees of Legendre polynomials to account for baseline drift, and the instructions subjects read during the scan were modeled in GLM as regressors of no interest. Motion parameters were used to compute the Euclidean norm (Enorm) of change in head position from one volume to the next. Volumes with Enorm values >.5 were censored from GLM. Clusters of activation data were warped to the MNI space prior to a region-of-interest (ROI) analysis (Evans et al., 1992).

*Regions of interest.* Primary data for the present analyses included brain responses from five distinct brain areas: right and left amygdala, right and left dlPFC, and vmPFC-sgACC. These ROIs were selected to characterize activity within neural structures involved with affective responding and cognitive control processes. Masks of these ROIs were then used to extract parameter estimates from the combat N-back fMRI task. Bilateral amygdala and vmPFC-sgACC ROIs were defined using the Harvard–Oxford MNI probabilistic atlas. ROIs for left dlPFC (−36, 44, 22) and right dlPFC (34, 44, 32) were defined as 5-mm spheres centered upon coordinates from a meta-analysis of fear conditioning (Fullana et al., 2016).

### 2.2. Data analysis

Analyses were conducted using a series of evolving mixed-effects multilevel path models in Mplus 6 (Muthén & Muthén, 2019). Analyses started from a basic task effect model to a task effect model moderated by CAPS and MN-BEST scores, before ending with a task effect model with tests of mediated moderation from CAPS and MN-BEST scores through personality dimensions. Participants were analyzed at level two with their neural activity for each of the four task conditions nested at level one. Predictor variables were z-scored before being included in the models to produce standardized betas as estimates, which can be interpreted as a measure of the size of the effect (Lorah, 2018).

*Model 1: Task effect model.* To examine the task effects on neural activity irrespective of individual differences measures, we estimated a mixed-effect multilevel path model predicting left/right amygdala, left/right dlPFC, and vmPFC-sgACC activity for each of the 0-back neutral, 2-back neutral, 0-back combat, 2-back combat blocks. To create a 2-by-2 factorial design, dummy variables were created for a cognitive load factor (0 = 0-back, 1 = 2-back) and an affect factor (0 = neutral, 1 = combat). Consequently, the 0-back neutral condition was included as the model intercept (β0) with additional fixed effects of cognitive load (β1), affect (β2), and cognitive load-by-affect interaction (β3). Individual variance components were also included by estimating random effects (intercept and slopes) for each of the predictors in the model.

*Model 2: Moderation model.* We tested for moderation as a function of individual differences in bmTBI severity and PTSS effects across task manipulations. Fixed effects from model 1 were included as well as MN-BEST blast severity, CAPS total severity scores, and their interaction as independent predictors and moderators of those fixed effects.

*Models 3a and 3b: Mediated moderation models.* Using multiply-mediated moderation, we modeled the degree to which bmTBI and PTSS moderation effects on neural responding could be explained by MMPI-2-RF H-O scores. Mediating variables included EID, THD, and BXD scale scores. When a significant direct effect was observed between MN-BEST or CAPS severity variables and an H-O scale, we planned to remove that particular H-O scale and substitute in its component RC scales in a separate, follow-up model (model 3b). Indirect effects for models 3a and 3b were estimated by testing the product of A and B paths. Partial-versus-full mediation was determined by examining whether or not the C’ path (i.e., the direct pathway of original moderator after co-varying for candidate mediators) remained significant following the identification of a significant mediator. Mediation models were also examined for non-traditional mediation effects such as complementary mediation, competitive mediation, indirect-only mediation, and direct-only mediation (Zhao et al., 2010). We applied family-wise Bonferroni correction to model 3b to control the type I error rate. Families were defined as groups of closely related questions, a list of which can be found in Table S16 of Supplemental Materials.

Parallel analyses using PSY-5-RF in place of H-O indices demonstrated convergent results. Given that the H-O scales were expandable by their RC scales, allowing for potentially greater descriptive resolution, we describe the results for H-O scales here, while PSY-5-RF analyses are detailed in Supplemental Materials (see Model S3).

## 3. Results

### 3.1. Demographics and clinical characteristics

Of the 93 participants (M_age_ = 34.35, SD_age_ = 8.36) analyzed within the fMRI models, 90 (96.77%) were male and 85 (91.40%) were non-Hispanic/white. Clinically, nine (9.68%) met DSM-IV-TR criteria for PTSD and not bmTBI; 25 (26.88%) met the criteria for bmTBI only and not PTSD; seven (7.53%) met the criteria for both PTSD and bmTBI; and 52 (55.91%) did not meet the criteria for either PTSD or bmTBI. However, of the latter group of 52, 17 had at least once met the criteria for PTSD in their lifetime, and nine met the criteria for subthreshold PTSD (i.e., meeting the criteria for some but not all PTSD symptom domains), indicating that these categories masked discernible impairments better detected with the CAPS dimensional symptom measure. The 52 who did not meet the criteria for either diagnosis exhibited average MN-BEST impact severity ratings of 2.17 (SD = 2.41) and current CAPS severity ratings of 21.83 (SD = 13.49), indicative of modest blast exposure and mild-moderate subthreshold PTSD symptomatology, on average. Descriptive statistics of CAPS and MN-BEST for the full sample are shown in Table 2.

**Table 2.** Sample statistics of CAPS and MN-BEST scores (*N* = 93)

	Mean (SD)	Observed range
**CAPS**		
** **Intrusions	7.55 (7.10)	0–26
** **Avoidance	2.95 (3.54)	0–15
** **Dysphoria	13.72 (10.82)	0–41
** **Hyperarousal	5.51 (3.64)	0–14
** **Total Severity	29.72 (21.04)	0–85
**MN-BEST**		
** **Months Since Blast^a^	81.49 (26.38)	26–133
** **Impact Severity Rating	1.97 (2.28)	0–9
** **Blast Severity Rating	1.17 (1.89)	0–8

*Note:* ^a^subset of individuals exposed to an explosive blast (*n* = 49); CAPS: Clinician Administered PTSD Scale, MN-BEST: Minnesota Blast Exposure Screening Tool

### 3.2. Task performance

A performance path model (Table S1) was first run to examine the effects of cognitive load and the effects of our affect manipulation using combat image content on performance indices (i.e., d-prime and reaction time). In sum, cognitive load significantly decreased d-prime (*β* = −0.504, *p* < .001) and increased reaction times (*β* = 0.471, *p* < .001), whereas combat images significantly interacted with the levels of cognitive load to predict d-prime (*β* = −0.471, *p* = .001) and reaction times (*β* = −0.23, *p* = .001). Re-estimating models at each level of cognitive load, simple effects analysis revealed that combat images decreased d-prime only under cognitive load (*β* = −0.298, SE = 0.094, *p* = .002, 95% CI = [−0.482, −0.114]), and increased reaction times only when cognitive load was absent (*β* = 0.357, SE = 0.073, *p* < .001, 95% CI = [0.213, 0.501]). We followed this with a performance moderation path model (Table S2) to examine the moderation of these effects by PTSS and bmTBI severity. In sum, PTSS increased the reaction times to combat images (*β* = 0.133, *p* = .018) and predicted significant changes in d-prime to cognitive load-by-affect interaction (*β* = −0.329, *p* = .008). Simple effects revealed that PTSS predicted decreased d-prime in the 0-back neutral condition (*β* = −0.459, SE = 0.193, *p* = .017, 95% CI = [−0.838, −0.081]), but not in the 2-back neutral condition (*β* = 0.052, SE = 0.216, *p* = .809, 95% CI = [−0.372, 0.477]). Performance indices failed to show moderation by bmTBI. Fuller statistical details of these models are provided in Table S3 of Supplemental Materials.

### 3.3. Effects of cognitive load and affective picture background (model 1)

**Figure 3.**
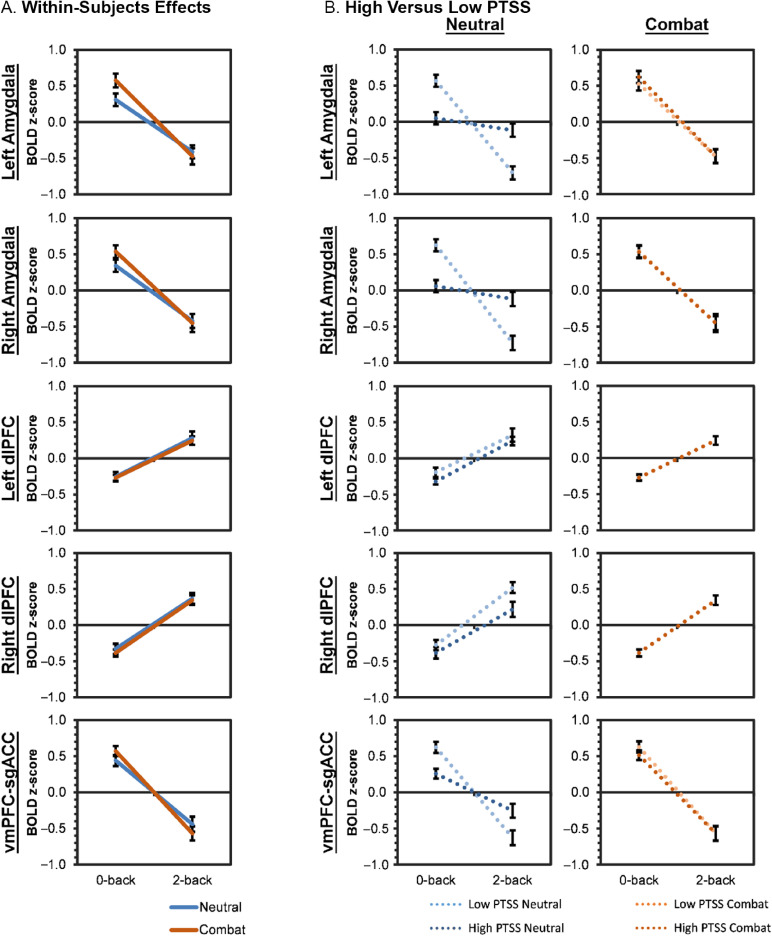
Model 2: PTSS and bilateral amygdala activity effects.

*Note:* Means and standard errors are displayed. (Panel A) Functional magnetic resonance imaging (fMRI) activations within regions of interest during cognitive load (0-back, 2-back) and affective (neutral, combat) manipulations. (Panel B) Moderation effects are displayed for individuals +1 and −1 standard deviations on posttraumatic stress disorder symptomatology (PTSS). PTSS moderated associations of left and right amygdala, and vmPFC-sgACC via blunted activation during the 0-back neutral condition. During the 2-back neutral condition, these same individuals with high PTSS also produced attenuated deactivations within the left and right amygdala. Additionally, high PTSS was associated with reduced right dlPFC activation during the 2-back condition. vmPFC = ventromedial prefrontal cortex, sgACC = subgenual anterior cingulate cortex, dlPFC = dorsolateral prefrontal cortex, BOLD = blood-oxygen-level-dependent.

**Figure 4.**
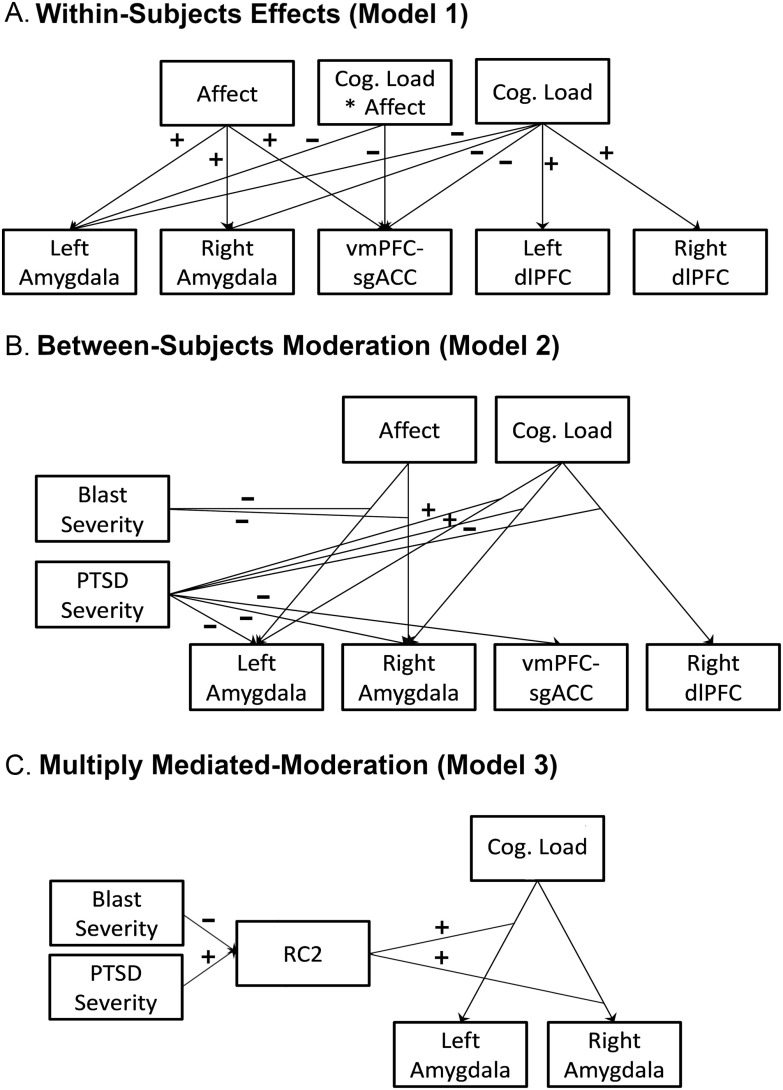
Statistical model effects.

*Note:* Simplified path diagram for the evolving mixed-effects multilevel path statistical models. (4A) Significant within-subject effects for the task manipulations are depicted. (4B) Only significant moderating effects of posttraumatic stress symptom and mild brain injury severity are depicted. (4C) Only significant paths to study effects via RC scales are shown. RC2 partially mediates the CAPS Severity moderation of bilateral amygdala under cognitive load. RCd indirectly and competitively mediates right amygdala activity under cognitive load. Cog. = 2-back neutral image condition predictor, vmPFC = ventromedial prefrontal cortex, sgACC = subgenual anterior cingulate cortex, dlPFC = dorsolateral prefrontal cortex, PTSD = posttraumatic stress disorder, RC2 = Low Positive Emotions.

Model 1 fixed and random effect estimates are displayed in Tables 3, S4, and S5 as well as Figures 3A and 4A. Bilateral amygdala (left: *β* = 0.307, *p* = .001; right: *β* = 0.339, *p* <.001) and vmPFC-sgACC (*β* = 0.438, *p* <.001) exhibited activations in the 0-back neutral condition. These activations became significant deactivations for bilateral amygdala (left: *β* = −0.717, *p* < .001; right: *β* = −0.761, *p* < .001) and vmPFC-sgACC (*β* = −0.879, *p* < .001) under cognitive load. Bilateral dlPFC exhibited deactivations in the 0-back neutral condition (left: *β* = −0.257, *p* < .001; right: *β* = −0.329, *p* < .001) that became significantly activated under cognitive load (left: *β* = 0.538, *p* < .001; right: *β* = 0.699, *p* < .001). From the 0-back neutral to 0-back combat condition, bilateral amygdala (left: *β* = 0.272, *p* = .005; right: *β* = 0.199, *p* = .020) and vmPFC-sgACC (*β* = 0.134, *p* = .049) exhibited increased activations, while bilateral dlPFC activation was not significantly changed (*p* > .263). Finally, cognitive load-by-affect interactions were found for left amygdala (*β* = −0.337, *p* = .002) and vmPFC-sgACC (*β* = −0.264, *p* = .006). Simple effect estimates for affect manipulation were derived by estimating models for 0-back and 2-back conditions separately. In the 0-back condition, combat images were associated with significant increases in left amygdala activity (*β* = 0.252, SE = 0.096, *p* = .009, 95% CI = [0.064, 0.439]) and marginal increases in vmPFC-sgACC activity (*β* = 0.122, SE = 0.068, *p* = .074, 95% CI = [−0.012, 0.256]). In the 2-back condition, combat images were no longer significantly associated with left amygdala activity (*β* = −0.047, *p* = .580) and was associated with marginally decreased vmPFC-sgACC activity (*β* = −0.120, SE = 0.072, *p* = .099, 95% CI = [−0.262, 0.022]).

**Table 3.** Model 1 Estimates of task effects by region of interest

Fixed effects	Left Amygdala	Right Amygdala	Left dlPFC	Right dlPFC	vmPFC-sgACC
No Load / intercept					
0-back neutral	.307**	.339***	−.257***	−.329***	.438***
Cognitive Load					
2-back neutral	−.717***	−.761***	.538***	.699***	−.879***
Affect					
0-back combat	.272**	.199*	−.011	−.055	.134*
Cognitive Load * Affect					
2-back combat	−.337**	−.232^†^	−.025	.030	−.264**

*Note:* Standardized beta values are displayed. 372 observations. ^†^ *p* < .10. *p < .05. **p < .01. ***p < .001. vmPFC = ventromedial prefrontal cortex, sgACC = subgenual anterior cingulate cortex, dlPFC = dorsolateral prefrontal cortex.

### 3.4. Moderation of brain activation by PTSD symptoms and history of bmTBI severity (model 2)

There were no significant interaction effects between PTSS and bmTBI severity, or between PTSS or bmTBI severity and the cognitive load-by-affect predictor for any ROI. There was also no significant association between PTSS and bmTBI severity. Therefore, all of these paths were removed from model 2 and analyses hereafter.

Estimates of model 2 fixed effects of PTSS and bmTBI severity to ROIs are displayed in Tables S6 and S7 and Figures 3B and 4B. Model 2 revealed that PTSS moderated bilateral amygdala, vmPFC-sgACC, and right dlPFC activity. Bilateral amygdala activity was also moderated by bmTBI. Specifically, greater PTSS was associated with less activity in bilateral amygdala (left: *β* = −0.259, *p* = .001; right: *β* = −0.283, *p* = .001) and vmPFC-sgACC (*β* = −0.182, *p* = .011) in the 0-back neutral condition, and was significantly associated with reduced bilateral amygdala deactivation (left: *β* = 0.295, *p* < .001; right: *β* = 0.304, *p* < .001) and right dlPFC activation (*β* = −0.150, *p* = .023) in the 2-back neutral condition. Greater bmTBI severity (while controlling for PTSS levels) was associated with reduced bilateral amygdala activity to combat images in the 0-back combat relative to the 0-back neutral condition (left: *β* = −0.201, *p* = .002; right: *β* = −0.205, *p* = .001).

### 3.5. Effect of personality on the relationship between PTSS and brain activations: Multiply-mediated moderation (models 3a and 3b)

Model 3a A-path fixed-effect estimates to H-O scales from PTSS and bmTBI are displayed in Tables S8 and S9, respectively. PTSS was associated with increased EID (*β* = 0.416, *p* < .001), while bmTBI severity was associated with decreased EID (*β* = −0.193, *p* = .022).

Estimates for B-path fixed effects from H-O scales to ROIs are displayed in Tables S10 and S11. These analyses revealed that greater EID was concurrently associated with significantly increased activity in bilateral amygdala under cognitive load (left: *β* = 0.220, *p* = .012; right: *β* = 0.185, *p* = .036) and increased activity in right dlPFC activity to combat images (*β* = 0.081, *p* = .020) over and above PTSS and bmTBI. Moreover, greater THD was associated with reduced vmPFC-sgACC activity in the 0-back neutral condition (*β* = −0.146, *p* = .028) and reduced left dlPFC activity to combat images (*β* = −0.053, *p* = .049) over and above PTSS and bmTBI.

Indirect effect estimates (shown from PTSS in Table S12 and from bmTBI in Table S13) showed that EID was found to significantly mediate PTSS-related cognitive load effects on left amygdala (*β* = 0.092, *p* = .041), and evidence that EID mediated the PTSS-related cognitive load effect on right amygdala was marginally significant (*β* = 0.077, *p* = .073).

Model 3a was re-estimated as model 3b after breaking EID into its component RC scales: RCd, RC2, and RC7. Analysis of A-paths (shown in Tables S8 and S9) revealed that increased PTSS was independently associated with increased RCd (*β* = 0.364, *p* < .001), RC2 (*β* = 0.387, *p* < .001), and RC7 (*β* = 0.393, *p* < .001) scores, while increased bmTBI severity was independently associated with decreased RCd (*β* = −0.199, *p* = .021) and RC2 (*β* = −0.272, *p* < .001) scores.

Analyses of B-paths (shown in Tables S10 and S11) revealed that RC2 was associated with increases in left (*β* = 0.372, *p* < .001) and right (*β* = 0.506, *p* < .001) amygdala activity under cognitive load over and above PTSS and bmTBI. B-paths were also initially found associating increased RCd with right amygdala deactivation under cognitive load, increased RC2 with vmPFC-sgACC activation under cognitive load, increased RC7 with vmPFC-sgACC activation under cognitive load, and increased RC7 with left amygdalar deactivation to combat images, but these paths did not survive Bonferroni correction (*p*’s > .085).

Indirect effect estimates revealed that RC2 was found to fully mediate PTSS-associated increases in left (*β* = 0.144, *p* = .004) and right (*β* = 0.196, *p* = .001) amygdala activity under cognitive load (Figure 4C and Table S12), as their corresponding C’ paths were not significant after Bonferroni correction (*p*’s > .205). RC2 was also found to be an indirect-only mediator from bmTBI severity to decreases in left (*β* = −0.101, *p* = .005) and right (*β* = −0.138, *p* = .002) amygdala activity under cognitive load (Figure 4C; Table S13). Indirect-only mediation was also initially found in PTSS to right amygdala deactivation under cognitive load through RCd, as well as PTSS to increased vmPFC-sgACC activity under cognitive load through both RC2 and RC7, but these paths did not survive Bonferroni correction (*p*’s > .080).

## 4. Discussion

We used an emotional N-back task to conduct a cross-sectional investigation examining how PTSS and bmTBI uniquely moderate neural activity in emotion regulation brain regions during increases in cognitive and affective demands. We then tested the patterns of neural activity for mediation by maladaptive personality traits characterized using the H-O scales from MMPI-2-RF (along with the PSY-5-RF scales, presented in Supplemental Materials). The emotion regulation network of bilateral amygdala, bilateral dlPFC, and vmPFC-sgACC generally responded to cognitive and affective demands as expected. Bilateral amygdala and vmPFC-sgACC activated to the threat cue, and dlPFC activated to increased working memory load. Amygdala and vmPFC-sgACC activity also decreased to increased working memory demands. Interestingly, the effect of PTSS was most evident in neural responses to low affective demands (i.e., neutral background images). Individuals with high levels of PTSS tended to not deactivate amygdala and vmPFC-sgACC regions, and have decreased right dlPFC activation under high cognitive load when neutral background images were presented. This appears to indicate that cognitive demand is not a factor in the responses of emotion regulation network for individuals with high PTSS. Thus, PTSS is associated with persistent emotion dysregulation when marshaling of cognitive resources is needed. Diminished right dlPFC increases may indicate a failure to tap these executive cognitive functions during more taxing tasks. It is also notable that when there were minimal cognitive and affective demands, high PTSS was associated with reduced amygdala and vmPFC-sgACC activity. One possibility is that PTSS was related to more relief by the absence of combat images in the 0-back condition, but that the stress of taxing cognitive demands in the 2-back condition limited reductions in amygdala and vmPFC-sgACC activity. Also, after taking into consideration PTSS, more severe bmTBI was associated with diminished amygdala activations to increased affective load, suggesting that bmTBI alone may have an opposing effect to PTSS on emotional reactivity.

We found that maladaptive personality mediated the relationship between PTSS and neural responses within the brain regions involved with emotional responding and regulation. EID mediated the abnormal amygdala responding under the 2-back neutral condition associated with PTSS. Subsequent modeling revealed that it was RC2 (low positive emotions), not RC7 (dysfunctional negative emotions) or RCd (demoralization), that partially mediated abnormal amygdala activity under the 2-back neutral condition, consistent with the idea that the persistent dysfunction and problems with societal reintegration are largely driven by emotional numbing, which limits the ability to derive pleasure from daily activities (i.e., anhedonia).

There were few mediational effects for other maladaptive personality traits on relationships between PTSS and neural responding during the emotional N-back task. Although PTSS did significantly correlate with NEGE-r, it was not as strong as the correlation with INTR-r. Furthermore, it was INTR-r, and not NEGE-r, that partially mediated amygdala abnormalities under the 2-back neutral condition associated with PTSS. PTSS was significantly associated with AGGR-r, but not BXD. Yet neither AGGR-r nor BXD scales mediated PTSS-associated neural abnormalities. Our findings diverged from Arbisi and colleagues (2011) who found that NEGE-r and RC7 were the primary indices separating individuals with PTSD from controls. We found that our PTSS-related amygdala activity abnormalities, controlling for bmTBI, were primarily indexed by INTR-r and RC2 instead. We did not detect any direct or indirect relations between PTSS or bmTBI with PSYC-r or THD, the two dimensions that would index disturbed perception. THD and PSYC-r were associated with decreased activity in vmPFC-sgACC activity under low cognitive and affective demands, and there was some unstable evidence suggesting THD was related to decreased left dlPFC under affective demands.

Meta-analyses have shown that elevated anxiety is reliably associated with reduced working memory performance in a domain-general manner, and this reduction is more pronounced in clinical samples (Moran, 2016). Intrusive thoughts and worry have also been shown to limit working memory capacity (Rosen & Engle, 1998). Findings of the current study of PTSS being associated with diminished dlPFC activation and smaller amygdala and vmPFC-sgACC deactivations during increased cognitive demands of the 2-back condition are consistent with internalizing psychopathology compromising working memory. Greater PTSS was also found to be associated with decreased activity in bilateral amygdala and vmPFC-sgACC under conditions of low cognitive and affective demand, which is inconsistent with expected hyperactivity, but may be reflective of compromised functioning. However, these effects were unexplained by personality indices.

A dimensional characterization of personality dysfunction may be useful for untangling commonly comorbid conditions like bmTBI and PTSD. The specific biological impact of mTBI in the context of a posttraumatic stress response remains largely unknown (Stein & McAllister, 2009; Vasterling et al., 2009). Therefore, the underlying nature of persistent and chronic symptoms is controversial, precisely because the physical damage produced by a bmTBI may alter brain function differently from the way emotional and psychological stress does (Ryan & Warden, 2003). We found that greater bmTBI, controlling for PTSS, was directly associated with reduced amygdala activity under affective load and indirectly associated with reduced amygdala activity under cognitive load. The indirect bmTBI effects on amygdala responses were mediated by reduced RC2 and INTR-r. Such effects are consistent with growing evidence that persistent bmTBI symptoms primarily reflect psychological factors rather than the direct concussive effects of injury, thereby contributing to psychiatric comorbidities of bmTBI (Hoge et al., 2009; Landre et al., 2006). Moreover, persistent mTBI symptoms have been consistently found to be more evident with an accompanying diagnosis of PTSD (Hoge et al., 2007; Schneiderman et al., 2008; Vanderploeg et al., 2009). To this point, Hoge and colleagues (2008) observed that PTSD was strongly related to mTBI, but after adjusting for PTSD and depression, mTBI was no longer associated with poor health and persisting symptoms. The present findings expand on this literature by showing that the neural impact of bmTBI is mediated by not just PTSS but more precisely by maladaptive personality factors represented by RC2 and INTR-r.

## 5. Strengths and limitations

There are several strengths of the current study. CAPS and MN-BEST are structured clinical interviews and yield dimensional measures and provide a thorough clinician-administered assessment of symptomatology. MMPI-2 RF is a comprehensively studied instrument for characterizing maladaptive personality traits and includes several validity scales to identify participants with valid responses. Affective and cognitive load conditions of the emotional N-back were designed as a laboratory analogue of threatening stimuli for US military veterans, and allowed us to effectively measure affective and cognitive processing under demanding conditions. A limitation is that participating veterans were 91% Caucasian and 97% male, and were recruited from individuals screened for mTBI at a Department of Veterans Affairs medical center who may or may not have been currently seeking treatment. As such, the findings may not generalize to all clinical settings or to more demographically diverse populations of veterans. It is also unclear whether the findings of this study would generalize to females given the nature of traumatic stressors that disproportionally affect women (e.g., sexual assault: Zoellner, Goodwin, & Foa, 2000) and intimate partner violence in women (Lang, Kennedy, & Stein, 2002). Also, the data presented are cross-sectional. Despite the path modeling and mediational statistical framework used, causal inferences should not be drawn from these analyses. Finally, because we limited the detection of BOLD signal to specific ROIs, it is likely that BOLD activity elsewhere in the brain may be explainable by additional PSY-5-RF and H-O variance.

## 6. Summary and future directions

The present study is the first fMRI investigation to test the mediating effects of maladaptive personality on abnormal responses in emotion regulation brain regions among individuals with PTSS and/or bmTBI under cognitive and affective challenge. PTSS was associated with decreased amygdala and vmPFC-sgACC activity during low cognitive and affective load, but under high cognitive load, these regions had comparatively greater activity in individuals with more PTSS. The effect of PTSS was partially mediated by maladaptive personality traits of anhedonia and introversion. Greater PTSS was also associated with diminished activation of dlPFC with increased cognitive demands, consistent with PTSS being associated with impaired use of cognitive resources when they are needed. We also found that increased bmTBI severity after taking into account the effect of PTSS was associated with decreased amygdala responding under affective load.

The results of this study represent a beginning effort to apply a personality neuroscience framework to the Hierarchical Taxonomy of Psychopathology (HiTOP) formulation of psychopathology (Perkins et al., 2019) with the goal of providing a more complete and mechanistic understanding of mental disorders such as PTSD. One aim of HiTOP is to clarify points of intersection and distinctiveness in psychopathology with the hope of untangling complex comorbidities due to within-disorder heterogeneity. In this study, we found that PTSS amygdala abnormality may represent a neural consequence relating to a central element of depression, mainly anhedonia. Noting that the activities within the ROIs reflecting the emotion regulatory system outlined in this investigation are generally correlated (see Tables S14 and S15 for estimated inter-region correlation statistics), future work should consider functional connectivity analysis as a way to understand how anhedonia perturbs the integrity and function of this system. Other neural abnormalities identified through the emotional N-back task were largely independent of depression or the severity of bmTBI. Anhedonia may be an important target for interventions intended to improve the affective and cognitive functioning of individuals with PTSD.

## Supplementary material

For supplementary material accompanying this paper visit http://dx.doi.org/10.1017/pen.2020.10. click here to view supplementary material
