# Supplementary material for: Posttraumatic stress symptomatology and abnormal neural responding during emotion regulation under cognitive demands: mediating effects of personality
Source: Personal Neurosci. 2020 Jul 30;3:e9. doi: 10.1017/pen.2020.10 (PMC7443821; doi:10.1017/pen.2020.10)
Supplement: Supplementary file 1 [file S2513988620000103sup001.docx]

**Posttraumatic Stress Symptomatology and Abnormal Neural Responding**

**during Emotion Regulation Under Cognitive Demands: Mediating Effects of Personality**

***Supplemental Materials***

**Task Performance**

Behavioral performance was assessed with a d-prime signal detection metric and reaction times. D-prime was selected for signal detection because hit and false alarm rates are jointly influenced by a subject’s sensitivity for signal vs. noise differentiation and their individual response biases (Stanislaw and Todorov, 1999). D-prime was computed from the following formula:

*D-prime* = Φ-1 (*H)* – Φ-1 *(F)*

Where Φ^-1^ (Inverse Phi) is a function that treats the response rates as probabilities and converts them into z-scores, and *H* is the hit rate, while *F* is the false alarm rate. Higher D-prime represents increased performance for detecting targets among non-targets. Reaction times (RT) were recorded during trials with correct identification of targets (i.e., hits). Responses occurring less than 150 ms after trial onset were excluded. Mean d-prime and RT were computed under each experimental condition. Given that RT typically exhibits positive skew (Tamm et al., 2012) , mean RTs were log-transformed. Both D-prime and log mean RT were then z-standardized before being input into models.

Task performance was assessed with a mixed-effects multilevel path model in Mplus 6. Participants were analyzed at level two with behavioral performance for each of the four task conditions nested at level one. Estimates were produced predicting d-prime and log RT for each of the 0-back neutral, 2-back neutral, 0-back combat, 2-back combat blocks. To create a 2-by-2 factorial design, dummy variables were created for a cognitive load factor (0 = 0-back, 1 = 2-back) and an affect factor (0 = neutral, 1 = combat). Consequently, the 0-back neutral condition was included as the model intercept (β_0_) with additional fixed effects of cognitive load (β_1_), affect (β_2_), and cognitive load-by-affect (β_3_). Individual variance components were also included by estimating random effects (intercept and slopes) for each of the predictors in the model.

Beta coefficients for the task performance model are displayed in Table S1, with fuller statistical details provided in Table S3. There was a significant cognitive load-by-affect interaction predicting d-prime (β = -0.471, p = .001) and log RT (β = -0.230, p = .001). Simple effects analysis via the re-estimation of effects at each level of cognitive load revealed that the affect manipulation predicted no effects on d-prime (p = 0.310) but slower RTs (β = 0.357, SE = 0.073, p < 0.001, 95% CI = [0.213, 0.501]) absent cognitive load, but predicted decreases in d-prime (β = -0.298, SE = 0.094, p = 0.002, 95% CI = [-0.482, -0.114]) and no effects on RT (p = 0.291) under cognitive load. There were also main effects of cognitive load that predicted significantly decreased d-prime (β = -0.504, p <.001) and slower RT (β = 0.471, p < .001).

A follow-up moderation model examined how PTSS and bmTBI moderated these effects. Fixed effects from the task performance model were included. Z-standardized CAPS total severity scores (PTSS), MN-BEST blast severity (bmTBI), and their interaction were also included as independent predictors and moderators of those fixed effects. There were no significant interaction effects between PTSS and bmTBI severity, or between PTSS or bmTBI severity and the cognitive load-by-affect predictor for d-prime. Therefore, terms for PTSS-by-bMTBI severity and PTSS and bmTBI severity pathways moderating the cognitive load * affect interaction on RT were removed and re-estimated.

Beta coefficients for this performance moderation model are displayed in Table S2, with fuller statistical details provided in Table S3. Results suggest that increases in PTSS predicted decreased d-prime in the 0-back neutral condition (β = -0.467 , p = .049) and slower RTs under the affect manipulation (β = 0.133, p = .018). PTSS also moderated the cognitive load-by-affect interaction on d-prime. Simple effects analysis via the re-estimation of effects at each level of cognitive load revealed that PTSS predicted decreased d-prime in the neutral condition absent cognitive load (β = -0.459, SE = 0.193, p = 0.017, 95% CI = [-0.838, -0.081]), but was not significantly associated with d-prime in the neutral condition under cognitive load (p = 0.809). BmTBI exhibited no significant moderation on performance indices.

| Table S1. *Performance* *model estimates of task effects by performance index* | | |
| --- | --- | --- |
| Fixed Effects | D-prime | Log RT |
| **No Load / intercept**  0-back neutral | **0.630*** | **-1.114***** |
| **Cognitive Load**  2-back neutral | **-0.504***** | **-0.471***** |
| **Affect**  0-back combat | 0.162 | **0.310***** |
| **Cognitive Load * Affect**  2-back combat | **-0.471**** | **-0.230^**^** |
| Variance Components |  |  |
| **No Load / intercept**  0-back neutral | 0.019 | **0.201*** |
| **Cognitive Load / slope**  2-back neutral | **0.146***** | **0.141*** |
| **Affect / slope**  0-back combat | 0.004 | **0.187***** |
| Cognitive Load * Affect / slope  2-back combat | 0.015 | 0.082^†^ |

Note: Standardized beta values are displayed. RT = Reaction Time. ^†^*p* < .10, **p* < .05, ***p* < .01, ****p* < .001.

| Table S2. *Performance indices moderated by PTSS and bmTBI* | | |
| --- | --- | --- |
| Fixed Effects | D-prime | Log RT |
| PTSS * |  |  |
| **No Load**  0-back neutral | **-0.467*** | 0.008 |
| **Cognitive Load**  2-back neutral | 0.080 | -0.052 |
| **Affect**  0-back combat | 0.172 | **0.133*** |
| **Cognitive Load * Affect**  2-back combat | **-0.329**** | **--** |
| bmTBI * |  |  |
| No Load  0-back neutral | 0.197 | 0.002 |
| Cognitive Load  2-back neutral | 0.041 | -0.122^†^ |
| Affect  0-back combat | -0.125 | 0.050 |
| Cognitive Load * Affect  2-back combat | 0.016 | **--** |

Note: Standardized beta values are displayed. RT = Reaction Time. ^†^*p* < .10, **p* < .05, ***p* < .01, ****p* < .001.

Table S3. *Statistical details of* *task effects on performance indices and their moderation by PTSS and bmTBI*

|  |  |  |  | 95% CI | |  |
| --- | --- | --- | --- | --- | --- | --- |
|  | β | SE(β) | *p* | Low | High | Variance Component |
| **Task Performance Model** |  |  |  |  |  |  |
| No Load (0-back Neutral) |  |  |  |  |  |  |
| **D-prime** | **0.630** | **0.259** | **.015*** | **0.122** | **1.138** | 0.019 |
| **Log RT** | **-1.114** | **0.131** | **<.001***** | **-1.370** | **-0.858** | **0.201*** |
| Cognitive Load (2-back Neutral) |  |  |  |  |  |  |
| **D-prime** | **-0.504** | **0.111** | **<.001***** | **-0.721** | **-0.286** | **0.146***** |
| **Log RT** | **0.471** | **0.071** | **<.001***** | **0.333** | **0.61** | **0.187***** |
| Affect (0-back Combat) |  |  |  |  |  |  |
| D-prime | 0.162 | 0.109 | .136 | -0.051 | 0.375 | 0.004 |
| **Log RT** | **0.310** | **0.064** | **<.001***** | **0.184** | **0.436** | **0.141*** |
| Cognitive Load * Affect (2-back Combat) |  |  |  |  |  |  |
| **D-prime** | **-0.471** | **0.142** | **.001**** | **-0.749** | **-0.193** | 0.015 |
| **Log RT** | **-0.230** | **0.072** | **.001**** | **-0.370** | **-0.089** | 0.082^†^ |
| D-prime 🡨🡪 Log RT | -0.032 | 0.018 | .067^†^ | -0.067 | 0.002 | *--* |
| **Task Performance Moderation Model** |  |  |  |  |  |  |
| PTSS * |  |  |  |  |  |  |
| No Load (0-back Neutral) |  |  |  |  |  |  |
| **D-prime** | **-0.467** | **0.237** | **.049*** | **-0.933** | **-0.002** | *--* |
| Log RT | 0.008 | 0.099 | .935 | -0.185 | 0.201 | *--* |
| Cognitive Load (2-back Neutral) |  |  |  |  |  |  |
| D-prime | 0.080 | 0.109 | .464 | -0.134 | 0.294 | *--* |
| Log RT | -0.052 | 0.058 | .363 | -0.165 | 0.060 | *--* |
| Affect (0-back Combat) |  |  |  |  |  |  |
| D-prime | 0.172 | 0.112 | .125 | -0.048 | 0.391 | *--* |
| **Log RT** | **0.133** | **0.057** | **.018** | **0.022** | **0.244** | *--* |
| Cognitive Load * Affect (2-back Combat) |  |  |  |  |  |  |
| **D-prime** | **-0.329** | **0.123** | **.008*** | **-0.571** | **-0.087** | *--* |
| Log RT | -- | -- | -- | -- | -- | *--* |
| bmTBI * |  |  |  |  |  |  |
| No Load (0-back Neutral) |  |  |  |  |  |  |
| D-prime | 0.197 | 0.189 | .299 | -0.174 | 0.567 | *--* |
| Log RT | 0.002 | 0.095 | .979 | -0.184 | 0.189 | *--* |
| Cognitive Load (2-back Neutral) |  |  |  |  |  |  |
| D-prime | 0.041 | 0.086 | .635 | -0.127 | 0.209 | *--* |
| Log RT | -0.122 | 0.066 | .066^†^ | -0.252 | 0.008 | *--* |
| Affect (0-back Combat) |  |  |  |  |  |  |
| D-prime | -0.125 | 0.082 | .127 | -0.286 | 0.036 | *--* |
| Log RT | 0.050 | 0.044 | .250 | -0.036 | 0.136 | *--* |
| Cognitive Load * Affect (2-back Combat) |  |  |  |  |  |  |
| D-prime | 0.016 | 0.096 | .870 | -0.172 | 0.204 | *--* |
| Log RT | -- | -- | -- | -- | -- | *--* |
| D-prime 🡨🡪 Log RT | -0.024 | 0.019 | .215 | -0.062 | 0.014 | -- |

Note: Predictors were z-scored prior to inclusion in the model. P-values shown here are not corrected for multiple comparisons. ^†^*p* < .10. **p* < .05. ***p* < .01. ****p* < .001.

**PSY-5-RF Scales as Mediators (Model S3)**

When PSY-5-RF scales were inserted as mediators for Model S3, there was no evidence for moderation of cognitive load-by-affect effects. Therefore, Model S3 was re-estimated after the mediational paths to the cognitive load-by-affect effects were removed. PTSS severity (Table S8) was independently associated with elevated Introversion/Low Positive Emotionality (INTR-r; β = 0.412, p < .001), Aggressiveness (AGGR-r; β = 0.202, p = .045), and Negative Emotionality/Neuroticism (NEGE-r β = 0.375, p < .001) scores, and greater bmTBI severity (Table S9) was independently associated with lower INTR-r scores (β = -0.223, p = .006).

After including the PSY-5-RF scales, the direct association between PTSS and vmPFC-sgACC activity in the 0-back neutral image condition was no longer significant (p = .073). However, PTSS remained associated with blunted bilateral amygdalar activity during 0-back neutral image trials (left: β = -0.210, p = .019; right: β = -0.283, p = .001). Also, PTSS remained associated with greater bilateral amygdalar activity (left: β = 0.194, p = .011; right: β = 0.304, p < .001) and attenuated right dlPFC (β = -0.150, p = .023) activity under cognitive load. The statistical details of all C’ paths from PTSS can be found in Table S12. BmTBI severity remained associated with decreased bilateral amygdalar activity under the affect manipulation (left: β = -0.213, p = .004; right: β = -0.225, p < .001).

When controlling for PTSS (and bmTBI), greater INTR-r scores were associated with more deactivation within bilateral amygdala under cognitive load (left: β = 0.221, p = .003; right: β = 0.228, p = .008). Also, greater Psychoticism (PSYC-r) scores were associated with decreased vmPFC-sgACC activity in the 0-back neutral condition (β = -0.155, p = .027). Beta-coefficients for all B-paths can be found in Table S10 and their statistical details can be found in Table S11.

Significant indirect effects were found from PTSS through INTR-r, which was consistent with partial mediated moderation of bilateral amygdalar task effects under cognitive load (left: β = 0.091, p = .022; right: β = 0.094, p = .023). INTR-r was also found to be a significant indirect-only mediator of bmTBI severity moderation of bilateral amygdalar activity under cognitive load (left: β = -0.049, p = .042; right: β = -0.051, p = .038). There was no evidence of additional PSY-5-RF indirect effects from PTSS or bmTBI. The statistical details of all indirect paths can be found in Table S12 for PTSS and Table S13 for bmTBI.

| Table S4. *Model 1 random effects estimates by region of interest* | | | | | |
| --- | --- | --- | --- | --- | --- |
| Variance Components | Left Amygdala | Right Amygdala | Left  dlPFC | Right dlPFC | vmPFC-sgACC |
| **No Load / intercept**  0-back Neutral | **0.114***** | **0.083*** | **0.290**** | **0.359***** | **0.183***** |
| **Cognitive Load / slope**  2-back Neutral | 0.052 | 0.050 | **0.616***** | **0.482***** | **0.329**** |
| Affective / slope  0-back Combat | 0.008 | 0.032 | 0.012 | 0.003 | 0.002 |
| Cognitive Load * Affect / slope  2-back Combat | 0.002 | 0.009 | 0.004 | 0.004 | 0.006 |

Note: Standardized beta values are displayed. 372 observations. **p* < .05, ***p* < .01, ****p* < .001.

Table S5. *Table for Model 1 for task effect estimates on each region of interest*

|  |  |  |  | 95% CI | |  |
| --- | --- | --- | --- | --- | --- | --- |
|  | β | SE(β) | *p* | Low | High | Variance Component |
| No Load (0-back Neutral / intercept) |  |  |  |  |  |  |
| **Left Amygdala** | **0.307** | **0.089** | **.001**** | **0.133** | **0.480** | **0.114***** |
| **Right Amygdala** | **0.339** | **0.089** | **<.001***** | **0.166** | **0.513** | **0.083*** |
| **Left dlPFC** | **-0.257** | **0.066** | **<.001***** | **-0.386** | **-0.128** | **0.290**** |
| **Right dlPFC** | **-0.329** | **0.071** | **<.001***** | **-0.469** | **-0.190** | **0.359***** |
| **vmPFC-sgACC** | **0.438** | **0.080** | **<.001***** | **0.281** | **0.595** | **0.183***** |
| Cognitive Load (2-back Neutral) |  |  |  |  |  |  |
| **Left Amygdala** | **-0.717** | **0.096** | **<.001***** | **-0.905** | **0.529** | 0.052 |
| **Right Amygdala** | **-0.761** | **0.102** | **<.001***** | **-0.962** | **-0.561** | 0.050 |
| **Left dlPFC** | **0.538** | **0.092** | **<.001***** | **0.357** | **0.718** | **0.616***** |
| **Right dlPFC** | **0.699** | **0.077** | **<.001***** | **0.547** | **0.850** | **0.482***** |
| **vmPFC-sgACC** | **-0.879** | **0.106** | **<.001***** | **-1.086** | **-0.672** | **0.329**** |
| Affect (0-back Combat) |  |  |  |  |  |  |
| **Left Amygdala** | **0.272** | **0.096** | **.005**** | **0.084** | **0.460** | 0.008 |
| **Right Amygdala** | **0.199** | **0.085** | **.020*** | **0.032** | **0.366** | 0.032 |
| Left dlPFC | -0.011 | 0.040 | .781 | -0.090 | 0.068 | 0.012 |
| Right dlPFC | -0.055 | 0.049 | .263 | -0.152 | 0.041 | 0.003 |
| **vmPFC-sgACC** | **0.134** | **0.068** | **.049*** | **0.000** | **0.268** | **0.329**** |
| Cognitive Load * Affect (2-back Combat) |  |  |  |  |  |  |
| **Left Amygdala** | **-0.337** | **0.111** | **.002**** | **-0.554** | **-0.120** | 0.002 |
| Right Amygdala | -0.232 | 0.126 | .066^†^ | -0.478 | 0.015 | 0.009 |
| Left dlPFC | -0.025 | 0.059 | .666 | -0.140 | 0.090 | 0.004 |
| Right dlPFC | 0.030 | 0.066 | .653 | -0.100 | 0.160 | 0.004 |
| **vmPFC-sgACC** | **-0.264** | **0.096** | **.006*** | **-0.453** | **-0.075** | 0.006 |

Note: Predictors were z-scored prior to inclusion in the model. P-values shown here are not corrected for multiple comparisons. ^†^*p* < .10. **p* < .05. ***p* < .01. ****p* < .001.

Table S6. *Model 2 estimates of brain activation moderation by PTSS and bmTBI severity*

| Model Effects | Left Amygdala | Right Amygdala | Left dlPFC | Right dlPFC | vmPFC-sgACC |
| --- | --- | --- | --- | --- | --- |
|  |  |  |  |  |  |
| PTSS * |  |  |  |  |  |
| **No Load** | **-0.259**** | **-0.283**** | -0.063 | -0.054 | **-0.182*** |
| 0-back Neutral |  |  |  |  |  |
| **Cognitive Load** | **0.295***** | **0.304***** | -0.041 | **-0.150*** | 0.186^†^ |
| 2-back Neutral |  |  |  |  |  |
| Affect | 0.048 | -0.005 | -0.003 | 0.001 | -0.057 |
| 0-back Combat |  |  |  |  |  |
| bmTBI * |  |  |  |  |  |
| No Load | 0.089 | 0.117 | 0.015 | -0.002 | -0.020 |
| 0-back Neutral |  |  |  |  |  |
| Cognitive Load | -0.079 | -0.140^†^ | 0.030 | 0.045 | -0.079 |
| 2-back Neutral |  |  |  |  |  |
| **Affect** | **-0.201**** | **-0.205***** | -0.027 | -0.026 | -0.044 |
| 0-back Combat |  |  |  |  |  |

Note: Model is displayed after pruning the non-significant interaction term with Cognitive Load-by-Affect. Correlational path between bmTBI severity and PTSS β = 0.196, SE = 0.116, p = 0.092, 95% CI = [-0.032, 0.423]. Predictors were z-scored prior to inclusion in the model. 372 observations. P-values shown here are not corrected for multiple comparisons. ^†^*p* < .10. **p* < .05. ***p* < .01. ****p* < .001.

Table S7. *Statistical details for PTSS and bmTBI moderation of task effects on each region of interest*

|  |  |  |  | 95% CI | |
| --- | --- | --- | --- | --- | --- |
|  | β | SE(β) | *p* | Low | High |
| PTSS * No Load (0-back Neutral / intercept) |  |  |  |  |  |
| **Left Amygdala** | **-0.259** | **0.075** | **.001**** | **-0.405** | **-0.113** |
| **Right Amygdala** | **-0.283** | **0.083** | **.001**** | **-0.445** | **-0.121** |
| Left dlPFC | -0.063 | 0.057 | .274 | -0.175 | 0.050 |
| Right dlPFC | -0.054 | 0.056 | .335 | -0.163 | 0.056 |
| **vmPFC-sgACC** | **-0.182** | **0.072** | **.011*** | **-0.323** | **-0.041** |
| PTSS * Cognitive Load (2-back Neutral / slope) |  |  |  |  |  |
| **Left Amygdala** | **0.295** | **0.072** | **<.001***** | **0.154** | **0.435** |
| **Right Amygdala** | **0.304** | **0.079** | **<.001***** | **0.150** | **0.459** |
| Left dlPFC | -0.041 | 0.082 | .620 | -0.202 | 0.121 |
| **Right dlPFC** | **-0.150** | **0.066** | **.023*** | **-0.280** | **-0.020** |
| vmPFC-sgACC | 0.186 | 0.099 | .059^†^ | -0.007 | 0.380 |
| PTSS * Affect (0-back Combat / slope) |  |  |  |  |  |
| Left Amygdala | 0.048 | 0.062 | .446 | -0.075 | 0.170 |
| Right Amygdala | -0.005 | 0.054 | .927 | -0.112 | 0.102 |
| Left dlPFC | -0.003 | 0.036 | .924 | -0.073 | 0.066 |
| Right dlPFC | 0.001 | 0.032 | .977 | -0.061 | 0.063 |
| vmPFC-sgACC | -0.057 | 0.041 | .166 | -0.137 | 0.024 |
| bmTBI * No Load (0-back Neutral / intercept) |  |  |  |  |  |
| Left Amygdala | 0.089 | 0.064 | .166 | -0.037 | 0.214 |
| Right Amygdala | 0.117 | 0.069 | .088^†^ | -0.017 | 0.252 |
| Left dlPFC | 0.015 | 0.092 | .868 | -0.165 | 0.195 |
| Right dlPFC | -0.002 | 0.077 | .983 | -0.152 | 0.149 |
| vmPFC-sgACC | -0.020 | 0.065 | .764 | -0.147 | 0.108 |
| bmTBI * Cognitive Load (2-back Neutral / slope) |  |  |  |  |  |
| Left Amygdala | -0.079 | 0.091 | .382 | -0.257 | 0.099 |
| Right Amygdala | -0.140 | 0.084 | .096^†^ | -0.304 | 0.025 |
| Left dlPFC | 0.030 | 0.11 | .786 | -0.185 | 0.245 |
| Right dlPFC | 0.045 | 0.072 | .533 | -0.096 | 0.186 |
| vmPFC-sgACC | -0.079 | 0.097 | .412 | -0.268 | 0.110 |
| bmTBI * Affect (0-back Combat / slope) |  |  |  |  |  |
| **Left Amygdala** | **-0.201** | **0.066** | **.002**** | **-0.331** | **-0.071** |
| **Right Amygdala** | **-0.205** | **0.061** | **.001**** | **-0.324** | **-0.085** |
| Left dlPFC | -0.027 | 0.029 | .361 | -0.085 | 0.031 |
| Right dlPFC | -0.026 | 0.037 | .490 | -0.098 | 0.047 |
| vmPFC-sgACC | -0.044 | 0.042 | .295 | -0.128 | 0.039 |

Note: Predictors were z-scored prior to inclusion in the model. P-values shown here are not corrected for multiple comparisons. Correlational path between bmTBI severity and PTSS β = 0.196, SE = 0.116, p = 0.092, 95% CI = [-0.032, 0.423].

Table S8. *PTSS* *predicting PSY-5-RF and H-O Scales across the multiply-mediated moderation models*

| CAPS Severity Predictor |  |  |  | 95% CI | |
| --- | --- | --- | --- | --- | --- |
|  | β | SE(β) | *p* | Low | High |
| PSY-5-RF^a^ |  |  |  |  |  |
| **INTR-r** | **0.412** | **0.097** | **< .001***** | **0.222** | **0.602** |
| **AGGR-r** | **0.202** | **0.101** | **.045*** | **0.005** | **0.399** |
| PSYC-r | 0.118 | 0.083 | .158 | -0.046 | 0.282 |
| DISC-r | 0.027 | 0.105 | .801 | -0.180 | 0.233 |
| **NEGE-r** | **0.375** | **0.096** | **< .001***** | **0.186** | **0.563** |
| H-O |  |  |  |  |  |
| **EID^b^** | **0.416** | **0.084** | **< .001***** | **0.252** | **0.580** |
| **RCd^c^** | **0.364** | **0.089** | **< .001***** | **0.189** | **0.539** |
| **RC2^c^** | **0.387** | **0.077** | **< .001***** | **0.237** | **0.538** |
| **RC7^c^** | **0.393** | **0.089** | **< .001***** | **0.219** | **0.568** |
| THD^b^ | 0.067 | 0.082 | .419 | -0.095 | 0.228 |
| BXD^b^ | 0.168 | 0.107 | .119 | -0.043 | 0.378 |

Note: Predictors were z-scored prior to inclusion in the model. ^a^Estimates from Model S3. ^b^Estimates from Model 3a. ^c^Estimates from Model 3b. **p* < .05, ***p* < .01, ****p* < .001.

Table S9. *BmTBI* s*everity predicting PSY-5-RF and H-O Scales across the multiply-mediated moderation models*

| BmTBI Severity Predictor |  |  |  | 95% CI | |
| --- | --- | --- | --- | --- | --- |
|  | β | SE(β) | *p* | Low | High |
| PSY-5-RF^a^ |  |  |  |  |  |
| **INTR-r** | **-0.223** | **0.081** | **.006**** | **-0.382** | **-0.063** |
| AGGR-r | 0.141 | 0.083 | .090^†^ | -0.022 | 0.304 |
| PSYC-r | -0.076 | 0.097 | .431 | -0.266 | 0.113 |
| DISC-r | -0.007 | 0.088 | .933 | -0.179 | 0.164 |
| NEGE-r | -0.119 | 0.097 | .218 | -0.308 | 0.070 |
| H-O |  |  |  |  |  |
| **EID^b^** | **-0.193** | **0.084** | **.022*** | **-0.357** | **-0.028** |
| **RCd^c^** | **-0.199** | **0.086** | **.021*** | **-0.367** | **-0.030** |
| **RC2^c^** | **-0.272** | **0.076** | **< .001***** | **-0.422** | **-0.123** |
| RC7^c^ | -0.148 | 0.095 | .119 | -0.334 | 0.038 |
| THD^b^ | -0.018 | 0.095 | .847 | -0.204 | 0.167 |
| BXD^b^ | -0.023 | 0.094 | .807 | -0.207 | 0.161 |

Note: Predictors were z-scored prior to inclusion in the model. ^a^Estimates from Model S3. ^b^Estimates from Model 3a. ^c^Estimates from Model 3b. ^†^*p* < .10, **p* < .05, ***p* < .01, ****p* < .001.

Table S10. *PSY-5-RF and H-O B-paths over and above CAPS and MN-BEST scores*

|  | Left Amygdala | Right Amygdala | Left dlPFC | Right dlPFC | vmPFC-sgACC |
| --- | --- | --- | --- | --- | --- |
| No Load |  |  |  |  |  |
| 0-back Neutral * |  |  |  |  |  |
| PSY-5-RF |  |  |  |  |  |
| INTR-r | -0.106 | -0.029 | -0.143^†^ | -0.032 | -0.059 |
| AGGR-r | -0.032 | -0.089 | -0.008 | -0.025 | -0.017 |
| **PSYC-r** | 0.078 | 0.031 | 0.092 | -0.132 | **-0.155*** |
| DISC-r | -0.082 | -0.044 | -0.014 | 0.077 | 0.019 |
| NEGE-r | -0.071 | -0.070 | 0.014 | 0.118 | -0.020 |
| H-O |  |  |  |  |  |
| EID ^a^ | -0.126 | -0.092 | -0.109^†^ | 0.051 | -0.095 |
| RCd ^b^ | -0.107 | 0.084 | -0.008 | 0.022 | 0.045 |
| RC2 ^b^ | -0.066 | -0.215^†^ | -0.158^†^ | -0.070 | -0.128 |
| RC7 ^b^ | -0.047 | -0.058 | -0.005 | 0.048 | -0.095 |
| **THD** ^a^ | -0.094 | -0.022 | 0.125^†^ | -0.090 | **-0.146*** |
| BXD ^a^ | -0.009 | -0.031 | 0.006 | 0.112^†^ | 0.070 |
| Cognitive Load |  |  |  |  |  |
| 2-back Neutral * |  |  |  |  |  |
| PSY-5-RF |  |  |  |  |  |
| **INTR-r** | **0.221**** | **0.228**** | 0.015 | 0.051 | 0.072 |
| AGGR-r | -0.053 | -0.059 | -0.034 | 0.022 | -0.040 |
| PSYC-r | 0.077 | 0.093 | 0.104 | -0.102 | 0.182^†^ |
| DISC-r | 0.057 | 0.076 | -0.046 | -0.013 | -0.111 |
| NEGE-r | 0.025 | 0.017 | -0.004 | 0.022 | 0.092 |
| H-O |  |  |  |  |  |
| **EID ^a^** | **0.220*** | **0.185*** | 0.041 | 0.074 | 0.170^†^ |
| **RCd ^b^** | -0.182 | **-0.371*** | -0.093 | 0.076 | -0.415^†^ |
| **RC2 ^b^** | **0.372***** | **0.506***** | 0.098 | 0.054 | **0.427*** |
| **RC7 ^b^** | 0.156 | 0.182 | 0.062 | -0.144 | **0.462**** |
| THD ^a^ | -0.031 | 0.018 | 0.109 | -0.046 | 0.090 |
| BXD ^a^ | 0.002 | 0.024 | -0.127^†^ | -0.019 | -0.125 |
| Affect |  |  |  |  |  |
| 0-back Combat * |  |  |  |  |  |
| PSY-5-RF |  |  |  |  |  |
| INTR-r | -0.010 | -0.030 | 0.048 | 0.028 | 0.025 |
| AGGR-r | -0.061 | -0.114^†^ | -0.042 | -0.016 | -0.050 |
| PSYC-r | -0.013 | -0.056 | -0.008 | -0.025 | 0.020 |
| DISC-r | 0.003 | 0.002 | 0.055 | 0.016 | 0.056 |
| NEGE-r | 0.058 | 0.043 | -0.044 | 0.017 | -0.008 |
| H-O |  |  |  |  |  |
| **EID ^a^** | 0.007 | -0.042 | 0.049 | **0.081*** | 0.025 |
| RCd ^b^ | 0.259^†^ | 0.130 | -0.043 | -0.010 | -0.012 |
| RC2 ^b^ | -0.125 | -0.074 | 0.086^†^ | 0.065 | 0.060 |
| **RC7 ^b^** | **-0.218*** | -0.163 | 0.013 | 0.037 | -0.038 |
| THD ^a^ | -0.017 | -0.021 | **-0.053*** | -0.045 | 0.005 |
| BXD ^a^ | 0.031 | 0.033 | 0.019 | 0.016 | 0.005 |

^a^Estimates from Model 3a. ^b^Estimates from Model 3b. ^†^*p* < .10. **p* < .05. ***p* < .01. ****p* < .001.

Table S11. *Statistical details of PSY-5-RF and H-O B-paths over and above CAPS and MN-BEST scores*

|  |  |  |  | 95% CI | |
| --- | --- | --- | --- | --- | --- |
|  | β | SE(β) | *p* | Low | High |
| 0-back Neutral |  |  |  |  |  |
| **Left Amygdala** |  |  |  |  |  |
| *PSY-5-RF Direct Effects* |  |  |  |  |  |
| INTR-r→LAMYG | -0.032 | 0.098 | .747 | -0.223 | 0.160 |
| AGGR-r→LAMYG | 0.078 | 0.093 | .399 | -0.104 | 0.260 |
| PSYC-r→LAMYG | -0.082 | 0.086 | .340 | -0.251 | 0.087 |
| DISC-r→LAMYG | -0.071 | 0.082 | .387 | -0.232 | 0.090 |
| NEGE-r→LAMYG | -0.106 | 0.096 | .266 | -0.293 | 0.081 |
| *H-O Direct Effects* |  |  |  |  |  |
| EID→LAMYG^a^ | -0.126 | 0.090 | .161 | -0.303 | 0.050 |
| RCd→LAMYG^b^ | -0.107 | 0.170 | .529 | -0.440 | 0.226 |
| RC2→LAMYG^b^ | -0.066 | 0.126 | .600 | -0.313 | 0.181 |
| RC7→LAMYG^b^ | -0.047 | 0.114 | .680 | -0.270 | 0.176 |
| THD→LAMYG^a^ | -0.094 | 0.078 | .232 | -0.247 | 0.060 |
| BXD→LAMYG^a^ | -0.009 | 0.072 | .902 | -0.150 | 0.132 |
| **Right Amygdala** |  |  |  |  |  |
| *PSY-5-RF Direct Effects* |  |  |  |  |  |
| INTR-r→RAMYG | -0.089 | 0.095 | .348 | -0.274 | 0.097 |
| AGGR-r→RAMYG | 0.031 | 0.088 | .728 | -0.142 | 0.203 |
| PSYC-r→RAMYG | -0.044 | 0.100 | .664 | -0.240 | 0.153 |
| DISC-r→RAMYG | -0.070 | 0.087 | .421 | -0.239 | 0.100 |
| NEGE-r→RAMYG | -0.029 | 0.109 | .793 | -0.243 | 0.186 |
| *H-O Direct Effects* |  |  |  |  |  |
| EID→RAMYG^a^ | -0.092 | 0.094 | .327 | -0.276 | 0.092 |
| RCd→RAMYG^b^ | 0.084 | 0.151 | .577 | -0.212 | 0.380 |
| RC2→RAMYG^b^ | -0.215 | 0.120 | .074^†^ | -0.450 | 0.021 |
| RC7→RAMYG^b^ | -0.058 | 0.129 | .651 | -0.310 | 0.194 |
| THD→RAMYG^a^ | -0.022 | 0.091 | .809 | -0.200 | 0.156 |
| BXD→RAMYG^a^ | -0.031 | 0.085 | .715 | -0.197 | 0.135 |
| **Left dlPFC** |  |  |  |  |  |
| *PSY-5-RF Direct Effects* |  |  |  |  |  |
| INTR-r→LDLPFC | -0.143 | 0.078 | .067^†^ | -0.296 | 0.010 |
| AGGR-r→LDLPFC | -0.008 | 0.076 | .913 | -0.157 | 0.141 |
| PSYC-r→LDLPFC | 0.092 | 0.067 | .175 | -0.041 | 0.224 |
| DISC-r→LDLPFC | -0.014 | 0.062 | .817 | -0.137 | 0.108 |
| NEGE-r→LDLPFC | 0.014 | 0.078 | .859 | -0.139 | 0.166 |
| *H-O Direct Effects* |  |  |  |  |  |
| EID→LDLPFC^a^ | -0.109 | 0.060 | .068^†^ | -0.226 | 0.008 |
| RCd→LDLPFC^b^ | -0.008 | 0.153 | .957 | -0.308 | 0.291 |
| RC2→LDLPFC^b^ | -0.158 | 0.091 | .082^†^ | -0.336 | 0.020 |
| RC7→LDLPFC^b^ | -0.005 | 0.107 | .959 | -0.214 | 0.203 |
| THD→LDLPFC^a^ | 0.125 | 0.069 | .069^†^ | -0.010 | 0.260 |
| BXD→LDLPFC^a^ | 0.006 | 0.062 | .924 | -0.115 | 0.127 |
| **Right dlPFC** |  |  |  |  |  |
| *PSY-5-RF Direct Effects* |  |  |  |  |  |
| INTR-r→RDLPFC | -0.032 | 0.060 | .591 | -0.149 | 0.085 |
| AGGR-r→RDLPFC | -0.025 | 0.058 | .668 | -0.139 | 0.089 |
| PSYC-r→RDLPFC | -0.132 | 0.085 | .118 | -0.299 | 0.034 |
| DISC-r→RDLPFC | 0.077 | 0.068 | .255 | -0.056 | 0.210 |
| NEGE-r→RDLPFC | 0.118 | 0.085 | .168 | -0.050 | 0.285 |
| *H-O Direct Effects* |  |  |  |  |  |
| EID→RDLPFC^a^ | 0.051 | 0.071 | .475 | -0.088 | 0.190 |
| RCd→RDLPFC^b^ | 0.022 | 0.139 | .876 | -0.250 | 0.294 |
| RC2→RDLPFC^b^ | -0.070 | 0.090 | .436 | -0.246 | 0.106 |
| RC7→RDLPFC^b^ | 0.048 | 0.111 | .663 | -0.169 | 0.265 |
| THD→RDLPFC^a^ | -0.090 | 0.069 | .193 | -0.226 | 0.046 |
| BXD→RDLPFC^a^ | 0.112 | 0.065 | .083^†^ | -0.015 | 0.238 |
| **vmPFC-sgACC** |  |  |  |  |  |
| *PSY-5-RF Direct Effects* |  |  |  |  |  |
| INTR-r→VMSG | -0.059 | 0.102 | .567 | -0.259 | 0.142 |
| AGGR-r→VMSG | -0.017 | 0.080 | .830 | -0.175 | 0.140 |
| **PSYC-r→VMSG** | **-0.155** | **0.070** | **.027*** | **-0.292** | **-0.018** |
| DISC-r→VMSG | 0.019 | 0.082 | .814 | -0.142 | 0.181 |
| NEGE-r→VMSG | -0.020 | 0.081 | .807 | -0.179 | 0.140 |
| *H-O Direct Effects* |  |  |  |  |  |
| EID→VMSG^a^ | -0.095 | 0.067 | .156 | -0.227 | 0.036 |
| RCd→VMSG^b^ | 0.045 | 0.157 | .775 | -0.262 | 0.351 |
| RC2→VMSG^b^ | -0.128 | 0.123 | .298 | -0.368 | 0.113 |
| RC7→VMSG^b^ | -0.095 | 0.122 | .435 | -0.334 | 0.144 |
| **THD→VMSG^a^** | **-0.146** | **0.066** | **.028*** | **-0.275** | **-0.016** |
| BXD→VMSG^a^ | 0.070 | 0.084 | .404 | -0.094 | 0.234 |
| 2-back Neutral |  |  |  |  |  |
| **Left Amygdala** |  |  |  |  |  |
| *PSY-5-RF Direct Effects* |  |  |  |  |  |
| **INTR-r→LAMYG** | **0.221** | **0.075** | **.003**** | **0.075** | **0.367** |
| AGGR-r→LAMYG | -0.053 | 0.078 | .493 | -0.205 | 0.099 |
| PSYC-r→LAMYG | 0.077 | 0.082 | .350 | -0.084 | 0.238 |
| DISC-r→LAMYG | 0.057 | 0.083 | .495 | -0.107 | 0.220 |
| NEGE-r→LAMYG | 0.025 | 0.086 | .766 | -0.142 | 0.193 |
| *H-O Direct Effects* |  |  |  |  |  |
| **EID→LAMYG^a^** | **0.220** | **0.088** | **.012*** | **0.048** | **0.393** |
| RCd→LAMYG^b^ | -0.182 | 0.131 | .166 | -0.440 | 0.075 |
| **RC2→LAMYG^b^** | **0.372** | **0.099** | **<.001***** | **0.178** | **0.567** |
| RC7→LAMYG^b^ | 0.156 | 0.106 | .140 | -0.051 | 0.364 |
| THD→LAMYG^a^ | -0.031 | 0.074 | .675 | -0.175 | 0.114 |
| BXD→LAMYG^a^ | 0.002 | 0.071 | .975 | -0.138 | 0.142 |
| **Right Amygdala** |  |  |  |  |  |
| *PSY-5-RF Direct Effects* |  |  |  |  |  |
| **INTR-r→RAMYG** | **0.228** | **0.085** | **.008**** | **0.061** | **0.396** |
| AGGR-r→RAMYG | -0.059 | 0.088 | .506 | -0.232 | 0.114 |
| PSYC-r→RAMYG | 0.093 | 0.064 | .144 | -0.032 | 0.219 |
| DISC-r→RAMYG | 0.076 | 0.085 | .372 | -0.091 | 0.243 |
| NEGE-r→RAMYG | 0.017 | 0.077 | .828 | -0.134 | 0.168 |
| *H-O Direct Effects* |  |  |  |  |  |
| **EID→RAMYG^a^** | **0.185** | **0.088** | **.036*** | **0.012** | **0.358** |
| **RCd→RAMYG^b^** | **-0.371** | **0.146** | **.011*** | **-0.657** | **-0.086** |
| **RC2→RAMYG^b^** | **0.506** | **0.107** | **<.001***** | **0.295** | **0.717** |
| RC7→RAMYG^b^ | 0.182 | 0.113 | .108 | -0.040 | 0.404 |
| THD→RAMYG^a^ | 0.018 | 0.071 | .800 | -0.121 | 0.156 |
| BXD→RAMYG^a^ | 0.024 | 0.073 | .745 | -0.119 | 0.167 |
| **Left dlPFC** |  |  |  |  |  |
| *PSY-5-RF Direct Effects* |  |  |  |  |  |
| INTR-r→LDLPFC | 0.015 | 0.109 | .893 | -0.199 | 0.228 |
| AGGR-r→LDLPFC | -0.034 | 0.082 | .682 | -0.195 | 0.128 |
| PSYC-r→LDLPFC | 0.104 | 0.120 | .386 | -0.131 | 0.339 |
| DISC-r→LDLPFC | -0.046 | 0.074 | .534 | -0.192 | 0.100 |
| NEGE-r→LDLPFC | -0.004 | 0.115 | .972 | -0.230 | 0.222 |
| *H-O Direct Effects* |  |  |  |  |  |
| EID→LDLPFC^a^ | 0.041 | 0.084 | .626 | -0.124 | 0.206 |
| RCd→LDLPFC^b^ | -0.093 | 0.166 | .576 | -0.418 | 0.233 |
| RC2→LDLPFC^b^ | 0.098 | 0.137 | .474 | -0.171 | 0.367 |
| RC7→LDLPFC^b^ | 0.062 | 0.131 | .637 | -0.196 | 0.320 |
| THD→LDLPFC^a^ | 0.109 | 0.096 | .253 | -0.078 | 0.297 |
| BXD→LDLPFC^a^ | -0.127 | 0.069 | .067^†^ | -0.262 | 0.009 |
| **Right dlPFC** |  |  |  |  |  |
| *PSY-5-RF Direct Effects* |  |  |  |  |  |
| INTR-r→RDLPFC | 0.051 | 0.082 | .533 | -0.109 | 0.211 |
| AGGR-r→RDLPFC | 0.022 | 0.067 | .742 | -0.109 | 0.152 |
| PSYC-r→RDLPFC | -0.102 | 0.086 | .234 | -0.270 | 0.066 |
| DISC-r→RDLPFC | -0.013 | 0.075 | .868 | -0.160 | 0.135 |
| NEGE-r→RDLPFC | 0.022 | 0.081 | .789 | -0.137 | 0.181 |
| *H-O Direct Effects* |  |  |  |  |  |
| EID→RDLPFC^a^ | 0.074 | 0.069 | .286 | -0.062 | 0.209 |
| RCd→RDLPFC^b^ | 0.076 | 0.165 | .644 | -0.246 | 0.399 |
| RC2→RDLPFC^b^ | 0.054 | 0.106 | .607 | -0.153 | 0.262 |
| RC7→RDLPFC^b^ | -0.144 | 0.129 | .266 | -0.396 | 0.109 |
| THD→RDLPFC^a^ | -0.046 | 0.080 | .567 | -0.202 | 0.111 |
| BXD→RDLPFC^a^ | -0.019 | 0.067 | .783 | -0.150 | 0.113 |
| **vmPFC-sgACC** |  |  |  |  |  |
| *PSY-5-RF Direct Effects* |  |  |  |  |  |
| INTR-r→VMSG | 0.072 | 0.126 | .572 | -0.176 | 0.319 |
| AGGR-r→VMSG | -0.040 | 0.119 | .737 | -0.274 | 0.194 |
| PSYC-r→VMSG | 0.182 | 0.103 | .079^†^ | -0.021 | 0.384 |
| DISC-r→VMSG | -0.111 | 0.140 | .427 | -0.385 | 0.163 |
| NEGE-r→VMSG | 0.092 | 0.106 | .385 | -0.116 | 0.301 |
| *H-O Direct Effects* |  |  |  |  |  |
| EID→VMSG^a^ | 0.170 | 0.094 | .071^†^ | -0.015 | 0.355 |
| RCd→VMSG^b^ | -0.415 | 0.236 | .078^†^ | -0.877 | 0.046 |
| **RC2→VMSG^b^** | **0.427** | **0.179** | **.017*** | **0.076** | **0.777** |
| **RC7→VMSG^b^** | **0.462** | **0.172** | **.007**** | **0.124** | **0.800** |
| THD→VMSG^a^ | 0.090 | 0.095 | .342 | -0.096 | 0.277 |
| BXD→VMSG^a^ | -0.125 | 0.134 | .352 | -0.387 | 0.138 |
| 0-back Combat |  |  |  |  |  |
| **Left Amygdala** |  |  |  |  |  |
| *PSY-5-RF Direct Effects* |  |  |  |  |  |
| INTR-r→LAMYG | -0.061 | 0.080 | .447 | -0.217 | 0.096 |
| AGGR-r→LAMYG | -0.013 | 0.072 | .857 | -0.154 | 0.128 |
| PSYC-r→LAMYG | 0.003 | 0.085 | .971 | -0.163 | 0.169 |
| DISC-r→LAMYG | 0.058 | 0.081 | .479 | -0.102 | 0.217 |
| NEGE-r→LAMYG | -0.010 | 0.089 | .908 | -0.185 | 0.164 |
| *H-O Direct Effects* |  |  |  |  |  |
| EID→LAMYG^a^ | 0.007 | 0.082 | .927 | -0.153 | 0.168 |
| RCd→LAMYG^b^ | 0.259 | 0.133 | .052^†^ | -0.003 | 0.520 |
| RC2→LAMYG^b^ | -0.125 | 0.095 | .191 | -0.311 | 0.062 |
| **RC7→LAMYG^b^** | **-0.218** | **0.104** | **.036*** | **-0.422** | **-0.014** |
| THD→LAMYG^a^ | -0.017 | 0.080 | .837 | -0.174 | 0.141 |
| BXD→LAMYG^a^ | 0.031 | 0.074 | .679 | -0.115 | 0.177 |
| **Right Amygdala** |  |  |  |  |  |
| *PSY-5-RF Direct Effects* |  |  |  |  |  |
| INTR-r→RAMYG | -0.114 | 0.068 | .093^†^ | -0.246 | 0.019 |
| AGGR-r→RAMYG | -0.056 | 0.063 | .375 | -0.180 | 0.068 |
| PSYC-r→RAMYG | 0.002 | 0.082 | .982 | -0.159 | 0.163 |
| DISC-r→RAMYG | 0.043 | 0.075 | .572 | -0.105 | 0.190 |
| NEGE-r→RAMYG | -0.030 | 0.074 | .686 | -0.176 | 0.116 |
| *H-O Direct Effects* |  |  |  |  |  |
| EID→RAMYG^a^ | -0.042 | 0.074 | .564 | -0.187 | 0.102 |
| RCd→RAMYG^b^ | 0.130 | 0.129 | .312 | -0.122 | 0.382 |
| RC2→RAMYG^b^ | -0.074 | 0.084 | .384 | -0.239 | 0.092 |
| RC7→RAMYG^b^ | -0.163 | 0.101 | .105 | -0.361 | 0.034 |
| THD→RAMYG^a^ | -0.021 | 0.080 | .792 | -0.179 | 0.136 |
| BXD→RAMYG^a^ | 0.033 | 0.078 | .673 | -0.120 | 0.187 |
| **Left dlPFC** |  |  |  |  |  |
| *PSY-5-RF Direct Effects* |  |  |  |  |  |
| INTR-r→LDLPFC | 0.048 | 0.037 | .199 | -0.025 | 0.121 |
| AGGR-r→LDLPFC | -0.042 | 0.032 | .190 | -0.105 | 0.021 |
| PSYC-r→LDLPFC | -0.008 | 0.033 | .816 | -0.072 | 0.057 |
| DISC-r→LDLPFC | 0.055 | 0.034 | .100 | -0.011 | 0.121 |
| NEGE-r→LDLPFC | -0.044 | 0.047 | .344 | -0.135 | 0.047 |
| *H-O Direct Effects* |  |  |  |  |  |
| EID→LDLPFC^a^ | 0.049 | 0.032 | .125 | -0.014 | 0.112 |
| RCd→LDLPFC^b^ | -0.043 | 0.066 | .517 | -0.172 | 0.087 |
| RC2→LDLPFC^b^ | 0.086 | 0.051 | .095^†^ | -0.015 | 0.187 |
| RC7→LDLPFC^b^ | 0.013 | 0.054 | .809 | -0.092 | 0.118 |
| **THD→LDLPFC^a^** | **-0.053** | **0.027** | **.049*** | **-0.105** | **0.000** |
| BXD→LDLPFC^a^ | 0.019 | 0.033 | .555 | -0.045 | 0.083 |
| **Right dlPFC** |  |  |  |  |  |
| *PSY-5-RF Direct Effects* |  |  |  |  |  |
| INTR-r→RDLPFC | 0.028 | 0.043 | .517 | -0.057 | 0.112 |
| AGGR-r→RDLPFC | -0.016 | 0.033 | .631 | -0.081 | 0.049 |
| PSYC-r→RDLPFC | -0.025 | 0.036 | .484 | -0.096 | 0.045 |
| DISC-r→RDLPFC | 0.016 | 0.033 | .620 | -0.048 | 0.081 |
| NEGE-r→RDLPFC | 0.017 | 0.048 | .724 | -0.077 | 0.111 |
| *H-O Direct Effects* |  |  |  |  |  |
| **EID→RDLPFC^a^** | **0.081** | **0.035** | **.020*** | **0.013** | **0.150** |
| RCd→RDLPFC^b^ | -0.010 | 0.070 | .882 | -0.148 | 0.127 |
| RC2→RDLPFC^b^ | 0.065 | 0.060 | .277 | -0.052 | 0.183 |
| RC7→RDLPFC^b^ | 0.037 | 0.066 | .574 | -0.093 | 0.167 |
| THD→RDLPFC^a^ | -0.045 | 0.032 | .154 | -0.107 | 0.017 |
| BXD→RDLPFC^a^ | 0.016 | 0.033 | .633 | -0.049 | 0.080 |
| **vmPFC-sgACC** |  |  |  |  |  |
| *PSY-5-RF Direct Effects* |  |  |  |  |  |
| INTR-r→VMSG | 0.025 | 0.066 | .708 | -0.105 | 0.154 |
| AGGR-r→VMSG | -0.050 | 0.051 | .326 | -0.149 | 0.050 |
| PSYC-r→VMSG | 0.020 | 0.047 | .667 | -0.072 | 0.113 |
| DISC-r→VMSG | 0.056 | 0.059 | .341 | -0.059 | 0.171 |
| NEGE-r→VMSG | -0.008 | 0.060 | .894 | -0.125 | 0.109 |
| *H-O Direct Effects* |  |  |  |  |  |
| EID→VMSG^a^ | 0.025 | 0.049 | .603 | -0.07 | 0.121 |
| RCd→VMSG^b^ | -0.012 | 0.094 | .895 | -0.196 | 0.171 |
| RC2→VMSG^b^ | 0.060 | 0.070 | .396 | -0.078 | 0.198 |
| RC7→VMSG^b^ | -0.038 | 0.075 | .612 | -0.186 | 0.109 |
| THD→VMSG^a^ | 0.005 | 0.047 | .914 | -0.087 | 0.097 |
| BXD→VMSG^a^ | 0.005 | 0.050 | .922 | -0.094 | 0.104 |

Note: Predictors were z-scored prior to inclusion in the model. Full set of multiple mediator estimates shown for significantly moderated paths from Model 2 only.  ^a^Estimates from Model 3a. ^b^Estimates from Model 3b. P-values shown here are not corrected for multiple comparisons. ^†^*p* < .10. **p* < .05. ***p* < .01. ****p* < .001.

Table S12. *Table of C paths, C’ paths, and indirect associations with PTSS*

|  |  |  |  | 95% CI | |
| --- | --- | --- | --- | --- | --- |
|  | β | SE(β) | *p* | Low | High |
| 0-back Neutral |  |  |  |  |  |
| **PTSS→Left Amygdala (C)** | **-0.259** | **0.075** | **.001**** | **-0.382** | **-0.136** |
| *PSY-5-RF Indirect Effects* |  |  |  |  |  |
| **C'** | **-0.210** | **0.090** | **.019*** | **-0.386** | **-0.034** |
| PTSS→INTR-r→LAMYG | -0.013 | 0.041 | .749 | -0.093 | 0.067 |
| PTSS→AGGR-r→LAMYG | 0.016 | 0.021 | .453 | -0.025 | 0.057 |
| PTSS→PSYC-r→LAMYG | -0.010 | 0.011 | .371 | -0.031 | 0.012 |
| PTSS→DISC-r→LAMYG | -0.002 | 0.008 | .813 | -0.017 | 0.014 |
| PTSS→NEGE-r→LAMYG | -0.040 | 0.038 | .301 | -0.115 | 0.036 |
| *H-O Indirect Effects* |  |  |  |  |  |
| **C'^a^** | **-0.199** | **0.084** | **.017*** | **-0.363** | **-0.035** |
| C'^b^ | -0.171 | 0.088 | .051^†^ | -0.343 | 0.001 |
| PTSS→EID→LAMYG^a^ | -0.052 | 0.041 | .203 | -0.133 | 0.028 |
| PTSS→RCd→LAMYG^b^ | -0.039 | 0.063 | .539 | -0.163 | 0.085 |
| PTSS→RC2→LAMYG^b^ | -0.026 | 0.050 | .607 | -0.123 | 0.072 |
| PTSS→RC7→LAMYG^b^ | -0.018 | 0.045 | .680 | -0.106 | 0.069 |
| PTSS→THD→LAMYG^a^ | -0.006 | 0.009 | .477 | -0.023 | 0.011 |
| PTSS→BXD→LAMYG^a^ | -0.001 | 0.012 | .904 | -0.025 | 0.022 |
| **PTSS→Right Amygdala (C)** | **-0.283** | **0.083** | **.001**** | **-0.419** | **-0.147** |
| *PSY-5-RF Indirect Effects* |  |  |  |  |  |
| **C'** | **-0.235** | **0.097** | **.016*** | **-0.425** | **-0.045** |
| PTSS→INTR-r→RAMYG | -0.037 | 0.040 | .356 | -0.114 | 0.041 |
| PTSS→AGGR-r→RAMYG | 0.006 | 0.018 | .735 | -0.029 | 0.042 |
| PTSS→PSYC-r→RAMYG | -0.005 | 0.012 | .669 | -0.029 | 0.018 |
| PTSS→DISC-r→RAMYG | -0.002 | 0.008 | .818 | -0.018 | 0.014 |
| PTSS→NEGE-r→RAMYG | -0.011 | 0.042 | .796 | -0.092 | 0.071 |
| *H-O Indirect Effects* |  |  |  |  |  |
| **C'^a^** | **-0.238** | **0.093** | **.010*** | **-0.420** | **-0.057** |
| **C'^b^** | **-0.197** | **0.091** | **.030*** | **-0.376** | **-0.019** |
| PTSS→EID→RAMYG^a^ | -0.038 | 0.042 | .359 | -0.120 | 0.043 |
| PTSS→RCd→RAMYG^b^ | 0.031 | 0.055 | .577 | -0.077 | 0.139 |
| PTSS→RC2→RAMYG^b^ | -0.083 | 0.051 | .100 | -0.182 | 0.016 |
| PTSS→RC7→RAMYG^b^ | -0.023 | 0.050 | .649 | -0.121 | 0.076 |
| PTSS→THD→RAMYG^a^ | -0.001 | 0.006 | .816 | -0.014 | 0.011 |
| PTSS→BXD→RAMYG^a^ | -0.005 | 0.015 | .738 | -0.035 | 0.025 |
| **PTSS→Left dlPFC (C)** | -0.063 | 0.057 | .274 | -0.157 | 0.032 |
| *PSY-5-RF Indirect Effects* |  |  |  |  |  |
| C' | -0.018 | 0.068 | .793 | -0.150 | 0.115 |
| PTSS→INTR-r→LDLPFC | -0.059 | 0.034 | .083^†^ | -0.126 | 0.008 |
| PTSS→AGGR-r→LDLPFC | -0.002 | 0.015 | .913 | -0.032 | 0.028 |
| PTSS→PSYC-r→LDLPFC | 0.011 | 0.011 | .312 | -0.010 | 0.032 |
| PTSS→DISC-r→LDLPFC | < 0.001 | 0.002 | .871 | -0.005 | 0.004 |
| PTSS→NEGE-r→LDLPFC | 0.005 | 0.029 | .859 | -0.052 | 0.063 |
| *H-O Indirect Effects* |  |  |  |  |  |
| C'^a^ | -0.027 | 0.063 | .675 | -0.151 | 0.098 |
| C'^b^ | -0.002 | 0.063 | .973 | -0.126 | 0.121 |
| PTSS→EID→LDLPFC^a^ | -0.045 | 0.028 | .103 | -0.100 | 0.009 |
| PTSS→RCd→LDLPFC^b^ | -0.003 | 0.056 | .957 | -0.112 | 0.106 |
| PTSS→RC2→LDLPFC^b^ | -0.061 | 0.037 | .102 | -0.135 | 0.012 |
| PTSS→RC7→LDLPFC^b^ | -0.002 | 0.042 | .959 | -0.084 | 0.080 |
| PTSS→THD→LDLPFC^a^ | 0.008 | 0.011 | .459 | -0.014 | 0.030 |
| PTSS→BXD→LDLPFC^a^ | 0.001 | 0.010 | .923 | -0.019 | 0.021 |
| **PTSS→Right dlPFC (C)** | -0.054 | 0.056 | .335 | -0.145 | 0.038 |
| *PSY-5-RF Indirect Effects* |  |  |  |  |  |
| C' | -0.066 | 0.054 | .218 | -0.171 | 0.039 |
| PTSS→INTR-r→RDLPFC | -0.013 | 0.025 | .595 | -0.062 | 0.035 |
| PTSS→AGGR-r→RDLPFC | -0.005 | 0.012 | .663 | -0.028 | 0.018 |
| PTSS→PSYC-r→RDLPFC | -0.016 | 0.016 | .331 | -0.047 | 0.016 |
| PTSS→DISC-r→RDLPFC | 0.002 | 0.008 | .805 | -0.014 | 0.018 |
| PTSS→NEGE-r→RDLPFC | 0.044 | 0.035 | .202 | -0.024 | 0.112 |
| *H-O Indirect Effects* |  |  |  |  |  |
| C'^a^ | -0.087 | 0.055 | .109 | -0.195 | 0.020 |
| C'^b^ | -0.064 | 0.059 | .280 | -0.181 | 0.052 |
| PTSS→EID→RDLPFC^a^ | 0.021 | 0.030 | .478 | -0.037 | 0.079 |
| PTSS→RCd→RDLPFC^b^ | 0.008 | 0.051 | .876 | -0.091 | 0.107 |
| PTSS→RC2→RDLPFC^b^ | -0.027 | 0.035 | .434 | -0.095 | 0.041 |
| PTSS→RC7→RDLPFC^b^ | 0.019 | 0.044 | .663 | -0.066 | 0.104 |
| PTSS→THD→RDLPFC^a^ | -0.006 | 0.009 | .499 | -0.023 | 0.011 |
| PTSS→BXD→RDLPFC^a^ | 0.019 | 0.015 | .220 | -0.011 | 0.049 |
| **PTSS→vmPFC-sgACC (C)** | **-0.223** | **0.081** | **.006**** | **-0.382** | **-0.063** |
| *PSY-5-RF Indirect Effects* |  |  |  |  |  |
| C' | -0.129 | 0.072 | .073^†^ | -0.270 | 0.012 |
| PTSS→INTR-r→VMSG | -0.024 | 0.043 | .571 | -0.108 | 0.060 |
| PTSS→AGGR-r→VMSG | -0.003 | 0.016 | .829 | -0.035 | 0.028 |
| PTSS→PSYC-r→VMSG | -0.018 | 0.016 | .260 | -0.050 | 0.014 |
| PTSS→DISC-r→VMSG | 0.001 | 0.003 | .855 | -0.005 | 0.006 |
| PTSS→NEGE-r→VMSG | -0.007 | 0.031 | .809 | -0.068 | 0.053 |
| *H-O Indirect Effects* |  |  |  |  |  |
| **C'^a^** | **-0.144** | **0.068** | **.034*** | **-0.278** | **-0.011** |
| C'^b^ | -0.113 | 0.069 | .100 | -0.248 | 0.022 |
| PTSS→EID→VMSG^a^ | -0.040 | 0.030 | .187 | -0.099 | 0.019 |
| PTSS→RCd→VMSG^b^ | 0.016 | 0.057 | .776 | -0.096 | 0.128 |
| PTSS→RC2→VMSG^b^ | -0.049 | 0.048 | .308 | -0.144 | 0.046 |
| PTSS→RC7→VMSG^b^ | -0.037 | 0.050 | .453 | -0.135 | 0.060 |
| PTSS→THD→VMSG^a^ | -0.010 | 0.013 | .445 | -0.035 | 0.015 |
| PTSS→BXD→VMSG^a^ | 0.012 | 0.014 | .393 | -0.015 | 0.039 |
| 2-back Neutral |  |  |  |  |  |
| **PTSS→Left Amygdala (C)** | **0.295** | **0.072** | **< .001***** | **0.177** | **0.412** |
| *PSY-5-RF Indirect Effects* |  |  |  |  |  |
| **C'** | **0.194** | **0.076** | **.011*** | **0.045** | **0.344** |
| **PTSS**→**INTR-r**→**LAMYG** | **0.091** | **0.040** | **.022*** | **0.013** | **0.169** |
| PTSS→AGGR-r→LAMYG | -0.011 | 0.016 | .502 | -0.042 | 0.021 |
| PTSS→PSYC-r→LAMYG | 0.009 | 0.011 | .400 | -0.012 | 0.030 |
| PTSS→DISC-r→LAMYG | 0.002 | 0.007 | .823 | -0.012 | 0.015 |
| PTSS→NEGE-r→LAMYG | 0.010 | 0.033 | .769 | -0.054 | 0.073 |
| *H-O Indirect Effects* |  |  |  |  |  |
| **C'^a^** | **0.205** | **0.079** | **.010*** | **0.049** | **0.361** |
| **C'^b^** | **0.150** | **0.073** | **.041*** | **0.006** | **0.293** |
| **PTSS**→**EID**→**LAMYG^a^** | **0.092** | **0.045** | **.041*** | **0.004** | **0.179** |
| PTSS→RCd→LAMYG^b^ | -0.066 | 0.049 | .175 | -0.162 | 0.030 |
| **PTSS**→**RC2**→**LAMYG^b^** | **0.144** | **0.050** | **.004**** | **0.046** | **0.242** |
| PTSS→RC7→LAMYG^b^ | 0.061 | 0.044 | .158 | -0.024 | 0.147 |
| PTSS→THD→LAMYG^a^ | -0.002 | 0.006 | .713 | -0.013 | 0.009 |
| PTSS→BXD→LAMYG^a^ | 00.00 | 0.012 | .975 | -0.023 | 0.024 |
| **PTSS→Right Amygdala (C)** | **0.304** | **0.079** | **< .001***** | **0.174** | **0.434** |
| *PSY-5-RF Indirect Effects* |  |  |  |  |  |
| **C'** | **0.203** | **0.087** | **.020*** | **0.032** | **0.373** |
| **PTSS**→**INTR-r**→**RAMYG** | **0.094** | **0.041** | **.023*** | **0.013** | **0.175** |
| PTSS→AGGR-r→RAMYG | -0.012 | 0.018 | .518 | -0.048 | 0.024 |
| PTSS→PSYC-r→RAMYG | 0.011 | 0.009 | .230 | -0.007 | 0.029 |
| PTSS→DISC-r→RAMYG | 0.002 | 0.009 | .814 | -0.015 | 0.019 |
| PTSS→NEGE-r→RAMYG | 0.006 | 0.029 | .830 | -0.051 | 0.063 |
| *H-O Indirect Effects* |  |  |  |  |  |
| **C'^a^** | **0.222** | **0.088** | **.012*** | **0.049** | **0.395** |
| C'^b^ | 0.153 | 0.080 | .054^†^ | -0.003 | 0.309 |
| PTSS→EID→RAMYG^a^ | 0.077 | 0.043 | .073^†^ | -0.007 | 0.161 |
| **PTSS**→**RCd**→**RAMYG^b^** | **-0.135** | **0.060** | **.024*** | **-0.252** | **-0.018** |
| **PTSS**→**RC2**→**RAMYG^b^** | **0.196** | **0.057** | **.001**** | **0.083** | **0.309** |
| PTSS→RC7→RAMYG^b^ | 0.072 | 0.045 | .109 | -0.016 | 0.159 |
| PTSS→THD→RAMYG^a^ | 0.001 | 0.005 | .808 | -0.008 | 0.011 |
| PTSS→BXD→RAMYG^a^ | 0.004 | 0.013 | .761 | -0.022 | 0.030 |
| **PTSS→Left dlPFC (C)** | -0.041 | 0.082 | .620 | -0.176 | 0.095 |
| *PSY-5-RF Indirect Effects* |  |  |  |  |  |
| C’ | -0.050 | 0.096 | .604 | -0.237 | 0.138 |
| PTSS→INTR-r→LDLPFC | 0.006 | 0.045 | .894 | -0.083 | 0.095 |
| PTSS→AGGR-r→LDLPFC | -0.007 | 0.017 | .686 | -0.040 | 0.026 |
| PTSS→PSYC-r→LDLPFC | 0.012 | 0.018 | .494 | -0.023 | 0.047 |
| PTSS→DISC-r→LDLPFC | -0.001 | 0.005 | .811 | -0.011 | 0.009 |
| PTSS→NEGE-r→LDLPFC | -0.002 | 0.043 | .972 | -0.086 | 0.083 |
| *H-O Indirect Effects* |  |  |  |  |  |
| C'^a^ | -0.044 | 0.088 | .615 | -0.216 | 0.128 |
| C'^b^ | -0.058 | 0.096 | .546 | -0.245 | 0.130 |
| PTSS→EID→LDLPFC^a^ | 0.017 | 0.035 | .625 | -0.051 | 0.085 |
| PTSS→RCd→LDLPFC^b^ | -0.034 | 0.063 | .590 | -0.156 | 0.089 |
| PTSS→RC2→LDLPFC^b^ | 0.038 | 0.055 | .486 | -0.069 | 0.145 |
| PTSS→RC7→LDLPFC^b^ | 0.024 | 0.053 | .643 | -0.079 | 0.128 |
| PTSS→THD→LDLPFC^a^ | 0.007 | 0.012 | .536 | -0.016 | 0.030 |
| PTSS→BXD→LDLPFC^a^ | -0.021 | 0.018 | .235 | -0.056 | 0.014 |
| **PTSS→Right dlPFC (C)** | **-0.150** | **0.066** | **.023*** | **-0.259** | **-0.041** |
| *PSY-5-RF Indirect Effects* |  |  |  |  |  |
| **C'** | **-0.171** | **0.072** | **.017*** | **-0.313** | **-0.030** |
| PTSS→INTR-r→RDLPFC | 0.051 | 0.082 | .533 | -0.109 | 0.211 |
| PTSS→AGGR-r→RDLPFC | 0.022 | 0.067 | .742 | -0.109 | 0.152 |
| PTSS→PSYC-r→RDLPFC | -0.102 | 0.086 | .234 | -0.270 | 0.066 |
| PTSS→DISC-r→RDLPFC | -0.013 | 0.075 | .868 | -0.160 | 0.135 |
| PTSS→NEGE-r→RDLPFC | 0.022 | 0.081 | .789 | -0.137 | 0.181 |
| *H-O Indirect Effects* |  |  |  |  |  |
| **C'^a^** | **-0.175** | **0.069** | **.011*** | **-0.309** | **-0.040** |
| **C'^b^** | **-0.144** | **0.069** | **.036*** | **-0.278** | **-0.010** |
| PTSS→EID→RDLPFC^a^ | 0.031 | 0.029 | .290 | -0.026 | 0.087 |
| PTSS→RCd→RDLPFC^b^ | 0.028 | 0.059 | .641 | -0.089 | 0.144 |
| PTSS→RC2→RDLPFC^b^ | 0.021 | 0.042 | .612 | -0.061 | 0.103 |
| PTSS→RC7→RDLPFC^b^ | -0.056 | 0.051 | .267 | -0.156 | 0.043 |
| PTSS→THD→RDLPFC^a^ | -0.003 | 0.006 | .632 | -0.016 | 0.009 |
| PTSS→BXD→RDLPFC^a^ | -0.003 | 0.011 | .787 | -0.026 | 0.019 |
| **PTSS→vmPFC-sgACC (C)** | 0.186 | 0.099 | .059^†^ | 0.024 | 0.348 |
| *PSY-5-RF Indirect Effects* |  |  |  |  |  |
| C' | 0.112 | 0.117 | .342 | -0.119 | 0.342 |
| PTSS→INTR-r→VMSG | 0.010 | 0.028 | .711 | -0.044 | 0.064 |
| PTSS→AGGR-r→VMSG | -0.010 | 0.012 | .417 | -0.034 | 0.014 |
| PTSS→PSYC-r→VMSG | 0.002 | 0.006 | .682 | -0.009 | 0.014 |
| PTSS→DISC-r→VMSG | 0.001 | 0.006 | .810 | -0.011 | 0.014 |
| PTSS→NEGE-r→VMSG | -0.003 | 0.022 | .894 | -0.047 | 0.041 |
| *H-O Indirect Effects* |  |  |  |  |  |
| C'^a^ | 0.130 | 0.112 | .244 | -0.089 | 0.349 |
| C'^b^ | -0.065 | 0.049 | .181 | -0.160 | 0.030 |
| PTSS→EID→VMSG^a^ | 0.071 | 0.042 | .089^†^ | -0.011 | 0.152 |
| PTSS→RCd→VMSG^b^ | -0.151 | 0.091 | .095^†^ | -0.329 | 0.027 |
| **PTSS→RC2→VMSG^b^** | **0.165** | **0.077** | **.032*** | **0.014** | **0.317** |
| **PTSS→RC7→VMSG^b^** | **0.182** | **0.075** | **.016*** | **0.034** | **0.330** |
| PTSS→THD→VMSG^a^ | 0.006 | 0.010 | .531 | -0.013 | 0.025 |
| PTSS→BXD→VMSG^a^ | -0.021 | 0.022 | .347 | -0.064 | 0.023 |
| 0-back Combat |  |  |  |  |  |
| **PTSS→Left Amygdala (C)** | 0.048 | 0.062 | .446 | -0.055 | 0.150 |
| *PSY-5-RF Indirect Effects* |  |  |  |  |  |
| C' | 0.077 | 0.073 | .290 | -0.066 | 0.220 |
| PTSS→INTR-r→LAMYG | -0.025 | 0.033 | .443 | -0.089 | 0.039 |
| PTSS→AGGR-r→LAMYG | -0.003 | 0.015 | .860 | -0.032 | 0.026 |
| PTSS→PSYC-r→LAMYG | < 0.001 | 0.010 | .970 | -0.019 | 0.020 |
| PTSS→DISC-r→LAMYG | 0.002 | 0.007 | .816 | -0.011 | 0.014 |
| PTSS→NEGE-r→LAMYG | -0.004 | 0.033 | .908 | -0.069 | 0.062 |
| *H-O Indirect Effects* |  |  |  |  |  |
| C'^a^ | 0.040 | 0.073 | .579 | -0.102 | 0.183 |
| C'^b^ | 0.082 | 0.072 | .253 | -0.059 | 0.223 |
| PTSS→EID→LAMYG^a^ | 0.003 | 0.034 | .928 | -0.064 | 0.070 |
| PTSS→RCd→LAMYG^b^ | 0.094 | 0.054 | .081^†^ | -0.012 | 0.200 |
| PTSS→RC2→LAMYGb | -0.048 | 0.037 | .191 | -0.121 | 0.024 |
| PTSS→RC7→LAMYG^b^ | -0.086 | 0.044 | .054^†^ | -0.173 | 0.001 |
| PTSS→THD→LAMYG^a^ | -0.001 | 0.006 | .847 | -0.012 | 0.010 |
| PTSS→BXD→LAMYG^a^ | 0.005 | 0.013 | .687 | -0.020 | 0.030 |
| **PTSS→Right Amygdala (C)** | -0.005 | 0.054 | .927 | -0.094 | 0.085 |
| *PSY-5-RF Indirect Effects* |  |  |  |  |  |
| C' | 0.063 | 0.060 | .294 | -0.055 | 0.181 |
| PTSS→INTR-r→RAMYG | -0.047 | 0.030 | .123 | -0.106 | 0.013 |
| PTSS→AGGR-r→RAMYG | -0.011 | 0.014 | .424 | -0.039 | 0.016 |
| PTSS→PSYC-r→RAMYG | < 0.001 | 0.010 | .982 | -0.019 | 0.019 |
| PTSS→DISC-r→RAMYG | 0.001 | 0.005 | .825 | -0.009 | 0.011 |
| PTSS→NEGE-r→RAMYG | -0.011 | 0.028 | .684 | -0.066 | 0.043 |
| *H-O Indirect Effects* |  |  |  |  |  |
| C'^a^ | 0.009 | 0.061 | .888 | -0.111 | 0.128 |
| C'^b^ | 0.034 | 0.062 | .580 | -0.087 | 0.156 |
| PTSS→EID→RAMYG^a^ | -0.018 | 0.031 | .563 | -0.077 | 0.042 |
| PTSS→RCd→RAMYG^b^ | 0.047 | 0.048 | .327 | -0.047 | 0.142 |
| PTSS→RC2→RAMYGb | -0.029 | 0.034 | .399 | -0.095 | 0.038 |
| PTSS→RC7→RAMYG^b^ | -0.064 | 0.043 | .138 | -0.149 | 0.021 |
| PTSS→THD→RAMYG^a^ | -0.001 | 0.006 | .802 | -0.012 | 0.010 |
| PTSS→BXD→RAMYG^a^ | 0.006 | 0.014 | .685 | -0.021 | 0.032 |
| **PTSS→Left dlPFC (C)** | -0.003 | 0.036 | .924 | -0.062 | 0.055 |
| *PSY-5-RF Indirect Effects* |  |  |  |  |  |
| C' | 0.001 | 0.035 | .969 | -0.067 | 0.070 |
| PTSS→INTR-r→LDLPFC | 0.020 | 0.016 | .230 | -0.012 | 0.052 |
| PTSS→AGGR-r→LDLPFC | -0.008 | 0.008 | .288 | -0.024 | 0.007 |
| PTSS→PSYC-r→LDLPFC | -0.001 | 0.004 | .817 | -0.009 | 0.007 |
| PTSS→DISC-r→LDLPFC | 0.001 | 0.006 | .809 | -0.010 | 0.013 |
| PTSS→NEGE-r→LDLPFC | -0.017 | 0.018 | .356 | -0.052 | 0.019 |
| *H-O Indirect Effects* |  |  |  |  |  |
| C'^a^ | -0.024 | 0.037 | .519 | -0.096 | 0.048 |
| C'^b^ | -0.029 | 0.040 | .464 | -0.106 | 0.048 |
| PTSS→EID→LDLPFC^a^ | 0.020 | 0.015 | .172 | -0.009 | 0.050 |
| PTSS→RCd→LDLPFC^b^ | -0.016 | 0.024 | .519 | -0.063 | 0.032 |
| PTSS→RC2→LDLPFCb | 0.033 | 0.021 | .115 | -0.008 | 0.075 |
| PTSS→RC7→LDLPFC^b^ | 0.005 | 0.021 | .809 | -0.036 | 0.047 |
| PTSS→THD→LDLPFC^a^ | -0.004 | 0.005 | .460 | -0.013 | 0.006 |
| PTSS→BXD→LDLPFC^a^ | 0.003 | 0.006 | .608 | -0.009 | 0.016 |
| PTSS→Right dlPFC (C) | 0.001 | 0.032 | .977 | -0.051 | 0.053 |
| *PSY-5-RF Indirect Effects* |  |  |  |  |  |
| C' | -0.011 | 0.038 | .767 | -0.085 | 0.063 |
| PTSS→INTR-r→RDLPFC | 0.011 | 0.018 | .520 | -0.024 | 0.047 |
| PTSS→AGGR-r→RDLPFC | -0.003 | 0.007 | .639 | -0.017 | 0.010 |
| PTSS→PSYC-r→RDLPFC | -0.003 | 0.005 | .555 | -0.013 | 0.007 |
| PTSS→DISC-r→RDLPFC | < 0.001 | 0.002 | .828 | -0.003 | 0.004 |
| PTSS→NEGE-r→RDLPFC | 0.006 | 0.018 | .728 | -0.029 | 0.042 |
| *H-O Indirect Effects* |  |  |  |  |  |
| C'^a^ | -0.033 | 0.035 | .351 | -0.101 | 0.036 |
| C'^b^ | -0.036 | 0.036 | .316 | -0.107 | 0.035 |
| PTSS→EID→RDLPFC^a^ | 0.034 | 0.018 | .056^†^ | -0.001 | 0.068 |
| PTSS→RCd→RDLPFC^b^ | -0.004 | 0.025 | .881 | -0.054 | 0.046 |
| PTSS→RC2→RDLPFC^b^ | 0.025 | 0.024 | .282 | -0.021 | 0.072 |
| PTSS→RC7→RDLPFC^b^ | 0.015 | 0.026 | .573 | -0.036 | 0.066 |
| PTSS→THD→RDLPFC^a^ | -0.003 | 0.005 | .508 | -0.012 | 0.006 |
| PTSS→BXD→RDLPFC^a^ | 0.003 | 0.006 | .661 | -0.009 | 0.014 |
| **PTSS→vmPFC-sgACC (C)** | -0.057 | 0.041 | .166 | -0.124 | 0.011 |
| *PSY-5-RF Indirect Effects* |  |  |  |  |  |
| C' | -0.058 | 0.053 | .273 | -0.161 | 0.045 |
| PTSS→INTR-r→VMSG | 0.010 | 0.028 | .711 | -0.044 | 0.064 |
| PTSS→AGGR-r→VMSG | -0.010 | 0.012 | .417 | -0.034 | 0.014 |
| PTSS→PSYC-r→VMSG | 0.002 | 0.006 | .682 | -0.009 | 0.014 |
| PTSS→DISC-r→VMSG | 0.001 | 0.006 | .810 | -0.011 | 0.014 |
| PTSS→NEGE-r→VMSG | -0.003 | 0.022 | .894 | -0.047 | 0.041 |
| *H-O Indirect Effects* |  |  |  |  |  |
| C'^a^ | 0.130 | 0.112 | .244 | -0.089 | 0.349 |
| C'^b^ | -0.065 | 0.049 | .181 | -0.160 | 0.030 |
| PTSS→EID→VMSG^a^ | 0.011 | 0.021 | .608 | -0.030 | 0.051 |
| PTSS→RCd→VMSG^b^ | 0.016 | 0.057 | .776 | -0.096 | 0.128 |
| PTSS→RC2→VMSG^b^ | -0.049 | 0.048 | .308 | -0.144 | 0.046 |
| PTSS→RC7→VMSG^b^ | -0.037 | 0.050 | .453 | -0.135 | 0.060 |
| PTSS→THD→VMSG^a^ | < 0.001 | 0.003 | .914 | -0.006 | 0.006 |
| PTSS→BXD→VMSG^a^ | 0.001 | 0.009 | .923 | -0.016 | 0.018 |

Note: Predictors were z-scored prior to inclusion in the model. Full set of multiple mediator estimates shown for significantly moderated paths from Model 2 only.  ^a^Estimates from Model 3a. ^b^Estimates from Model 3b. P-values shown here are uncorrected. ^†^*p* < .10. **p* < .05. ***p* < .01. ****p* < .001.

Table S13. *Table of C paths, C’ paths, and indirect associations with bmTBI*

|  |  |  |  | 95% CI | |
| --- | --- | --- | --- | --- | --- |
|  | β | SE(β) | *p* | Low | High |
| 0-back Neutral |  |  |  |  |  |
| **bmTBI severity→Left Amygdala (C)** | 0.089 | 0.064 | .166 | -0.017 | 0.194 |
| *PSY-5-RF Indirect Effects* |  |  |  |  |  |
| C' | 0.051 | 0.077 | .504 | -0.099 | 0.201 |
| bmTBI severity→INTR-r→LAMYG | 0.007 | 0.022 | .746 | -0.036 | 0.050 |
| bmTBI severity→AGGR-r→LAMYG | 0.011 | 0.014 | .422 | -0.016 | 0.038 |
| bmTBI severity→PSYC-r→LAMYG | 0.006 | 0.011 | .553 | -0.014 | 0.027 |
| bmTBI severity→DISC-r→LAMYG | 0.001 | 0.006 | .933 | -0.012 | 0.013 |
| bmTBI severity→NEGE-r→LAMYG | 0.013 | 0.015 | .404 | -0.017 | 0.042 |
| H-O *Indirect Effects* |  |  |  |  |  |
| C'^a^ | 0.062 | 0.074 | .399 | -0.083 | 0.208 |
| C'^b^ | 0.041 | 0.079 | .603 | -0.114 | 0.196 |
| bmTBI severity→EID→LAMYG^a^ | 0.024 | 0.020 | .226 | -0.015 | 0.064 |
| bmTBI severity→RCd→LAMYG^b^ | 0.021 | 0.035 | .546 | -0.048 | 0.090 |
| bmTBI severity→RC2→LAMYG^b^ | 0.018 | 0.034 | .594 | -0.048 | 0.084 |
| bmTBI severity→RC7→LAMYG^b^ | 0.007 | 0.017 | .690 | -0.027 | 0.041 |
| bmTBI severity→THD→LAMYG^a^ | 0.002 | 0.009 | .850 | -0.016 | 0.019 |
| bmTBI severity→BXD→LAMYG^a^ | 0.000 | 0.002 | .910 | -0.003 | 0.004 |
| **bmTBI severity→Right Amygdala (C)** | 0.117 | 0.069 | .088^†^ | 0.004 | 0.230 |
| *PSY-5-RF Indirect Effects* |  |  |  |  |  |
| C' | 0.086 | 0.081 | .291 | -0.073 | 0.245 |
| bmTBI severity→INTR-r→RAMYG | 0.020 | 0.021 | .353 | -0.022 | 0.062 |
| bmTBI severity→AGGR-r→RAMYG | 0.004 | 0.012 | .727 | -0.020 | 0.028 |
| bmTBI severity→PSYC-r→RAMYG | 0.003 | 0.009 | .717 | -0.015 | 0.021 |
| bmTBI severity→DISC-r→RAMYG | 0.001 | 0.006 | .934 | -0.012 | 0.013 |
| bmTBI severity→NEGE-r→RAMYG | 0.003 | 0.013 | .796 | -0.023 | 0.029 |
| H-O *Indirect Effects* |  |  |  |  |  |
| C'^a^ | 0.098 | 0.078 | .208 | -0.055 | 0.252 |
| C'^b^ | 0.065 | 0.080 | .414 | -0.091 | 0.221 |
| bmTBI severity→EID→RAMYG^a^ | 0.018 | 0.019 | .363 | -0.020 | 0.056 |
| bmTBI severity→RCd→RAMYG^b^ | -0.017 | 0.030 | .583 | -0.076 | 0.043 |
| bmTBI severity→RC2→RAMYG^b^ | 0.059 | 0.033 | .075^†^ | -0.006 | 0.123 |
| bmTBI severity→RC7→RAMYG^b^ | 0.009 | 0.020 | .659 | -0.030 | 0.047 |
| bmTBI severity→THD→RAMYG^a^ | 0.000 | 0.003 | .890 | -0.005 | 0.006 |
| bmTBI severity→BXD→RAMYG^a^ | 0.001 | 0.004 | .847 | -0.006 | 0.008 |
| **bmTBI severity→Left dlPFC (C)** | 0.015 | 0.092 | .868 | -0.136 | 0.166 |
| *PSY-5-RF Indirect Effects* |  |  |  |  |  |
| C' | -0.007 | 0.094 | .941 | -0.190 | 0.176 |
| bmTBI severity→INTR-r→LDLPFC | 0.032 | 0.020 | .104 | -0.007 | 0.070 |
| bmTBI severity→AGGR-r→LDLPFC | -0.001 | 0.011 | .914 | -0.022 | 0.020 |
| bmTBI severity→PSYC-r→LDLPFC | -0.007 | 0.010 | .485 | -0.027 | 0.013 |
| bmTBI severity→DISC-r→LDLPFC | 0.000 | 0.001 | .938 | -0.003 | 0.003 |
| bmTBI severity→NEGE-r→LDLPFC | -0.002 | 0.009 | .859 | -0.020 | 0.017 |
| *H-O Indirect Effects* |  |  |  |  |  |
| C'^a^ | -0.003 | 0.092 | .971 | -0.183 | 0.177 |
| C'^b^ | -0.029 | 0.097 | .770 | -0.219 | 0.162 |
| bmTBI severity→EID→LDLPFC^a^ | 0.021 | 0.015 | .157 | -0.008 | 0.050 |
| bmTBI severity→RCd→LDLPFC^b^ | 0.002 | 0.030 | .957 | -0.058 | 0.061 |
| bmTBI severity→RC2→LDLPFC^b^ | 0.043 | 0.026 | .095^†^ | -0.008 | 0.094 |
| bmTBI severity→RC7→LDLPFC^b^ | 0.001 | 0.016 | .959 | -0.030 | 0.032 |
| bmTBI severity→THD→LDLPFC^a^ | -0.002 | 0.012 | .847 | -0.025 | 0.021 |
| bmTBI severity→BXD→LDLPFC^a^ | 0.000 | 0.002 | .931 | -0.003 | 0.003 |
| **bmTBI severity→Right dlPFC (C)** | -0.002 | 0.077 | .983 | -0.128 | 0.125 |
| *PSY-5-RF Indirect Effects* |  |  |  |  |  |
| C' | -0.001 | 0.083 | .993 | -0.163 | 0.161 |
| bmTBI severity→INTR-r→RDLPFC | 0.007 | 0.014 | .603 | -0.020 | 0.034 |
| bmTBI severity→AGGR-r→RDLPFC | -0.004 | 0.008 | .670 | -0.020 | 0.013 |
| bmTBI severity→PSYC-r→RDLPFC | 0.010 | 0.015 | .486 | -0.018 | 0.039 |
| bmTBI severity→DISC-r→RDLPFC | -0.001 | 0.007 | .932 | -0.014 | 0.012 |
| bmTBI severity→NEGE-r→RDLPFC | -0.014 | 0.016 | .375 | -0.045 | 0.017 |
| *H-O Indirect Effects* |  |  |  |  |  |
| C'^a^ | 0.009 | 0.077 | .906 | -0.141 | 0.159 |
| C'^b^ | -0.009 | 0.081 | .914 | -0.168 | 0.151 |
| bmTBI severity→EID→RDLPFC^a^ | -0.010 | 0.015 | .501 | -0.038 | 0.019 |
| bmTBI severity→RCd→RDLPFC^b^ | -0.004 | 0.028 | .876 | -0.058 | 0.050 |
| bmTBI severity→RC2→RDLPFC^b^ | 0.019 | 0.025 | .445 | -0.030 | 0.068 |
| bmTBI severity→RC7→RDLPFC^b^ | -0.007 | 0.017 | .678 | -0.041 | 0.027 |
| bmTBI severity→THD→RDLPFC^a^ | 0.002 | 0.009 | .853 | -0.016 | 0.019 |
| bmTBI severity→BXD→RDLPFC^a^ | -0.003 | 0.010 | .803 | -0.023 | 0.018 |
| **bmTBI severity→vmPFC-sgACC (C)** | -0.020 | 0.065 | .764 | -0.127 | 0.088 |
| *PSY-5-RF Indirect Effects* |  |  |  |  |  |
| C' | -0.044 | 0.069 | .524 | -0.180 | 0.092 |
| bmTBI severity→INTR-r→VMSG | 0.013 | 0.023 | .572 | -0.032 | 0.058 |
| bmTBI severity→AGGR-r→VMSG | -0.002 | 0.011 | .831 | -0.025 | 0.020 |
| bmTBI severity→PSYC-r→VMSG | 0.012 | 0.016 | .456 | -0.019 | 0.043 |
| bmTBI severity→DISC-r→VMSG | 0.000 | 0.002 | .935 | -0.004 | 0.003 |
| bmTBI severity→NEGE-r→VMSG | 0.002 | 0.010 | .806 | -0.017 | 0.021 |
| *H-O Indirect Effects* |  |  |  |  |  |
| C'^a^ | -0.039 | 0.068 | .567 | -0.172 | 0.094 |
| C'^b^ | -0.060 | 0.069 | .378 | -0.195 | 0.074 |
| bmTBI severity→EID→VMSG^a^ | 0.018 | 0.015 | .224 | -0.011 | 0.048 |
| bmTBI severity→RCd→VMSG^b^ | -0.009 | 0.031 | .775 | -0.070 | 0.052 |
| bmTBI severity→RC2→VMSG^b^ | 0.035 | 0.034 | .308 | -0.032 | 0.101 |
| bmTBI severity→RC7→VMSG^b^ | 0.014 | 0.020 | .487 | -0.026 | 0.054 |
| bmTBI severity→THD→VMSG^a^ | 0.003 | 0.014 | .848 | -0.025 | 0.030 |
| bmTBI severity→BXD→VMSG^a^ | -0.002 | 0.007 | .811 | -0.015 | 0.012 |
| 2-back Neutral |  |  |  |  |  |
| **bmTBI severity→Left Amygdala (C)** | -0.079 | 0.091 | .382 | -0.229 | 0.070 |
| *PSY-5-RF Indirect Effects* |  |  |  |  |  |
| C' | -0.013 | 0.087 | .879 | -0.185 | 0.158 |
| **bmTBI severity**→**INTR-r**→**LAMYG** | **-0.049** | **0.024** | **.042** | **-0.097** | **-0.002** |
| bmTBI severity→AGGR-r→LAMYG | -0.007 | 0.011 | .510 | -0.030 | 0.015 |
| bmTBI severity→PSYC-r→LAMYG | -0.006 | 0.010 | .546 | -0.025 | 0.013 |
| bmTBI severity→DISC-r→LAMYG | 0.000 | 0.005 | .933 | -0.010 | 0.009 |
| bmTBI severity→NEGE-r→LAMYG | -0.003 | 0.011 | .777 | -0.024 | 0.018 |
| *H-O Indirect Effects* |  |  |  |  |  |
| C'^a^ | -0.037 | 0.087 | .667 | -0.208 | 0.133 |
| C'^b^ | 0.010 | 0.079 | .901 | -0.146 | 0.166 |
| bmTBI severity→EID→LAMYG^a^ | -0.042 | 0.025 | .096 | -0.092 | 0.007 |
| bmTBI severity→RCd→LAMYG^b^ | 0.036 | 0.030 | .233 | -0.023 | 0.096 |
| **bmTBI severity**→**RC2**→**LAMYG^b^** | **-0.101** | **0.036** | **.005**** | **-0.172** | **-0.031** |
| bmTBI severity→RC7→LAMYG^b^ | -0.023 | 0.022 | .300 | -0.067 | 0.021 |
| bmTBI severity→THD→LAMYG^a^ | 0.001 | 0.003 | .869 | -0.006 | 0.007 |
| bmTBI severity→BXD→LAMYG^a^ | 0.000 | 0.002 | .975 | -0.003 | 0.003 |
| **bmTBI severity→Right Amygdala (C)** | -0.140 | 0.084 | .096^†^ | -0.277 | -0.002 |
| *PSY-5-RF Indirect Effects* |  |  |  |  |  |
| C' | -0.071 | 0.085 | .404 | -0.236 | 0.095 |
| **bmTBI severity**→**INTR-r**→**RAMYG** | **-0.051** | **0.025** | **.038*** | **-0.099** | **-0.003** |
| bmTBI severity→AGGR-r→RAMYG | -0.008 | 0.012 | .507 | -0.033 | 0.016 |
| bmTBI severity→PSYC-r→RAMYG | -0.007 | 0.010 | .495 | -0.028 | 0.013 |
| bmTBI severity→DISC-r→RAMYG | -0.001 | 0.007 | .933 | -0.014 | 0.012 |
| bmTBI severity→NEGE-r→RAMYG | -0.002 | 0.009 | .831 | -0.020 | 0.016 |
| *H-O Indirect Effects* |  |  |  |  |  |
| C'^a^ | -0.103 | 0.084 | .219 | -0.268 | 0.061 |
| C'^b^ | -0.045 | 0.074 | .543 | -0.191 | 0.101 |
| bmTBI severity→EID→RAMYG^a^ | -0.036 | 0.023 | .129 | -0.082 | 0.010 |
| bmTBI severity→RCd→RAMYG^b^ | 0.074 | 0.043 | .086^†^ | -0.010 | 0.158 |
| **bmTBI severity**→**RC2**→**RAMYG^b^** | **-0.138** | **0.045** | **.002**** | **-0.212** | **-0.064** |
| bmTBI severity→RC7→RAMYG^b^ | -0.027 | 0.024 | .256 | -0.073 | 0.020 |
| bmTBI severity→THD→RAMYG^a^ | 0.000 | 0.002 | .873 | -0.004 | 0.004 |
| bmTBI severity→BXD→RAMYG^a^ | -0.001 | 0.003 | .844 | -0.006 | 0.005 |
| **bmTBI severity→Left dlPFC (C)** | 0.030 | 0.110 | .786 | -0.151 | 0.210 |
| *PSY-5-RF Indirect Effects* |  |  |  |  |  |
| C’ | 0.045 | 0.116 | .697 | -0.182 | 0.272 |
| bmTBI severity→INTR-r→LDLPFC | -0.003 | 0.025 | .894 | -0.051 | 0.045 |
| bmTBI severity→AGGR-r→LDLPFC | -0.005 | 0.012 | .685 | -0.028 | 0.018 |
| bmTBI severity→PSYC-r→LDLPFC | -0.008 | 0.013 | .528 | -0.033 | 0.017 |
| bmTBI severity→DISC-r→LDLPFC | 0.000 | 0.004 | .932 | -0.008 | 0.008 |
| bmTBI severity→NEGE-r→LDLPFC | 0.000 | 0.014 | .972 | -0.026 | 0.027 |
| *H-O Indirect Effects* |  |  |  |  |  |
| C'^a^ | 0.037 | 0.107 | .731 | -0.174 | 0.247 |
| C'^b^ | 0.047 | 0.113 | .680 | -0.175 | 0.269 |
| bmTBI severity→EID→LDLPFC^a^ | -0.008 | 0.017 | .634 | -0.040 | 0.025 |
| bmTBI severity→RCd→LDLPFC^b^ | 0.018 | 0.035 | .597 | -0.050 | 0.087 |
| bmTBI severity→RC2→LDLPFC^b^ | -0.027 | 0.039 | .489 | -0.103 | 0.049 |
| bmTBI severity→RC7→LDLPFC^b^ | -0.009 | 0.020 | .652 | -0.049 | 0.031 |
| bmTBI severity→THD→LDLPFC^a^ | -0.002 | 0.010 | .843 | -0.022 | 0.018 |
| bmTBI severity→BXD→LDLPFC^a^ | 0.003 | 0.012 | .802 | -0.020 | 0.026 |
| **bmTBI severity→Right dlPFC (C)** | 0.045 | 0.072 | .533 | -0.073 | 0.163 |
| *PSY-5-RF Indirect Effects* |  |  |  |  |  |
| C' | 0.048 | 0.077 | .534 | -0.103 | 0.199 |
| bmTBI severity→INTR-r→RDLPFC | -0.011 | 0.019 | .551 | -0.049 | 0.026 |
| bmTBI severity→AGGR-r→RDLPFC | 0.003 | 0.010 | .753 | -0.016 | 0.022 |
| bmTBI severity→PSYC-r→RDLPFC | 0.008 | 0.012 | .531 | -0.017 | 0.032 |
| bmTBI severity→DISC-r→RDLPFC | 0.000 | 0.001 | .940 | -0.002 | 0.002 |
| bmTBI severity→NEGE-r→RDLPFC | -0.003 | 0.010 | .793 | -0.022 | 0.017 |
| *H-O Indirect Effects* |  |  |  |  |  |
| C'^a^ | 0.058 | 0.074 | .438 | -0.088 | 0.204 |
| C'^b^ | 0.054 | 0.073 | .458 | -0.088 | 0.196 |
| bmTBI severity→EID→RDLPFC^a^ | -0.014 | 0.015 | .333 | -0.043 | 0.015 |
| bmTBI severity→RCd→RDLPFC^b^ | -0.015 | 0.033 | .646 | -0.079 | 0.049 |
| bmTBI severity→RC2→RDLPFC^b^ | -0.015 | 0.029 | .611 | -0.072 | 0.042 |
| bmTBI severity→RC7→RDLPFC^b^ | 0.021 | 0.023 | .361 | -0.024 | 0.067 |
| bmTBI severity→THD→RDLPFC^a^ | 0.001 | 0.005 | .862 | -0.009 | 0.010 |
| bmTBI severity→BXD→RDLPFC^a^ | 0.000 | 0.002 | .854 | -0.004 | 0.005 |
| **bmTBI severity→vmPFC-sgACC (C)** | -0.079 | 0.097 | .412 | -0.238 | 0.080 |
| *PSY-5-RF Indirect Effects* |  |  |  |  |  |
| C' | -0.034 | 0.097 | .729 | -0.223 | 0.156 |
| bmTBI severity→INTR-r→VMSG | -0.016 | 0.028 | .568 | -0.071 | 0.039 |
| bmTBI severity→AGGR-r→VMSG | -0.006 | 0.017 | .737 | -0.039 | 0.027 |
| bmTBI severity→PSYC-r→VMSG | -0.014 | 0.020 | .485 | -0.053 | 0.025 |
| bmTBI severity→DISC-r→VMSG | 0.001 | 0.010 | .933 | -0.018 | 0.020 |
| bmTBI severity→NEGE-r→VMSG | -0.011 | 0.016 | .486 | -0.042 | 0.020 |
| *H-O Indirect Effects* |  |  |  |  |  |
| C'^a^ | -0.048 | 0.092 | .607 | -0.229 | 0.134 |
| C'^b^ | 0.021 | 0.091 | .818 | -0.158 | 0.200 |
| bmTBI severity→EID→VMSG^a^ | -0.033 | 0.023 | .161 | -0.079 | 0.013 |
| bmTBI severity→RCd→VMSG^b^ | 0.083 | 0.060 | .167 | -0.035 | 0.200 |
| bmTBI severity→RC2→VMSG^b^ | -0.116 | 0.059 | .050^†^ | -0.233 | 0.000 |
| bmTBI severity→RC7→VMSG^b^ | -0.068 | 0.051 | .179 | -0.168 | 0.031 |
| bmTBI severity→THD→VMSG^a^ | -0.002 | 0.009 | .851 | -0.019 | 0.016 |
| bmTBI severity→BXD→VMSG^a^ | 0.003 | 0.012 | .813 | -0.021 | 0.027 |
| 0-back Combat |  |  |  |  |  |
| **bmTBI severity→Left Amygdala (C)** | **-0.201** | **0.066** | **.002**** | **-0.310** | **-0.092** |
| *PSY-5-RF Indirect Effects* |  |  |  |  |  |
| **C'** | **-0.213** | **0.073** | **.004**** | **-0.357** | **-0.070** |
| bmTBI severity→INTR-r→LAMYG | 0.014 | 0.018 | .446 | -0.021 | 0.048 |
| bmTBI severity→AGGR-r→LAMYG | -0.002 | 0.010 | .857 | -0.022 | 0.018 |
| bmTBI severity→PSYC-r→LAMYG | < 0.001 | 0.007 | .971 | -0.013 | 0.013 |
| bmTBI severity→DISC-r→LAMYG | < 0.001 | 0.005 | .934 | -0.011 | 0.010 |
| bmTBI severity→NEGE-r→LAMYG | 0.001 | 0.011 | .908 | -0.020 | 0.022 |
| *H-O Indirect Effects* |  |  |  |  |  |
| **C'^a^** | **-0.199** | **0.072** | **.006**** | **-0.340** | **-0.058** |
| **C'^b^** | **-0.215** | **0.070** | **.002**** | **-0.352** | **-0.078** |
| bmTBI severity→EID→LAMYG^a^ | -0.001 | 0.016 | .928 | -0.032 | 0.029 |
| bmTBI severity→RCd→LAMYG^b^ | -0.051 | 0.034 | .135 | -0.119 | 0.016 |
| bmTBI severity→RC2→LAMYGb | 0.034 | 0.027 | .210 | -0.019 | 0.087 |
| bmTBI severity→RC7→LAMYG^b^ | 0.032 | 0.025 | .200 | -0.017 | 0.082 |
| bmTBI severity→THD→LAMYG^a^ | 0.000 | 0.002 | .859 | -0.003 | 0.004 |
| bmTBI severity→BXD→LAMYG^a^ | -0.001 | 0.003 | .833 | -0.007 | 0.006 |
| **bmTBI severity→Right Amygdala (C)** | **-0.205** | **0.061** | **.001**** | **-0.305** | **-0.104** |
| *PSY-5-RF Indirect Effects* |  |  |  |  |  |
| **C'** | **-0.225** | **0.064** | **< .001***** | **-0.351** | **-0.099** |
| bmTBI severity→INTR-r→RAMYG | 0.025 | 0.017 | .139 | -0.008 | 0.059 |
| bmTBI severity→AGGR-r→RAMYG | -0.008 | 0.010 | .415 | -0.027 | 0.011 |
| bmTBI severity→PSYC-r→RAMYG | < 0.001 | 0.006 | .982 | -0.013 | 0.012 |
| bmTBI severity→DISC-r→RAMYG | < 0.001 | 0.004 | .936 | -0.008 | 0.007 |
| bmTBI severity→NEGE-r→RAMYG | 0.004 | 0.009 | .692 | -0.014 | 0.021 |
| *H-O Indirect Effects* |  |  |  |  |  |
| **C'^a^** | **-0.212** | **0.064** | **.001**** | **-0.338** | **-0.087** |
| **C'^b^** | **-0.222** | **0.064** | **< .001***** | **-0.347** | **-0.097** |
| bmTBI severity→EID→RAMYG^a^ | 0.008 | 0.015 | .576 | -0.021 | 0.037 |
| bmTBI severity→RCd→RAMYG^b^ | -0.026 | 0.028 | .354 | -0.080 | 0.029 |
| bmTBI severity→RC2→RAMYGb | 0.020 | 0.024 | .396 | -0.026 | 0.066 |
| bmTBI severity→RC7→RAMYG^b^ | 0.024 | 0.021 | .240 | -0.016 | 0.065 |
| bmTBI severity→THD→RAMYG^a^ | 0.000 | 0.002 | .847 | -0.004 | 0.004 |
| bmTBI severity→BXD→RAMYG^a^ | -0.001 | 0.004 | .842 | -0.008 | 0.007 |
| **bmTBI severity→Left dlPFC (C)** | -0.027 | 0.029 | .361 | -0.075 | 0.022 |
| *PSY-5-RF Indirect Effects* |  |  |  |  |  |
| C' | -0.016 | 0.030 | .604 | -0.075 | 0.044 |
| bmTBI severity→INTR-r→LDLPFC | -0.011 | 0.010 | .278 | -0.030 | 0.009 |
| bmTBI severity→AGGR-r→LDLPFC | -0.006 | 0.006 | .300 | -0.017 | 0.005 |
| bmTBI severity→PSYC-r→LDLPFC | 0.001 | 0.003 | .820 | -0.004 | 0.006 |
| bmTBI severity→DISC-r→LDLPFC | < 0.001 | 0.005 | .933 | -0.010 | 0.009 |
| bmTBI severity→NEGE-r→LDLPFC | 0.005 | 0.007 | .437 | -0.008 | 0.019 |
| *H-O Indirect Effects* |  |  |  |  |  |
| C'^a^ | -0.018 | 0.030 | .552 | -0.077 | 0.041 |
| C'^b^ | -0.010 | 0.032 | .757 | -0.073 | 0.053 |
| bmTBI severity→EID→LDLPFC^a^ | -0.009 | 0.007 | .199 | -0.024 | 0.005 |
| bmTBI severity→RCd→LDLPFC^b^ | 0.008 | 0.014 | .535 | -0.018 | 0.035 |
| bmTBI severity→RC2→LDLPFCb | -0.023 | 0.016 | .143 | -0.055 | 0.008 |
| bmTBI severity→RC7→LDLPFC^b^ | -0.002 | 0.008 | .813 | -0.018 | 0.014 |
| bmTBI severity→THD→LDLPFC^a^ | 0.001 | 0.005 | .846 | -0.009 | 0.011 |
| bmTBI severity→BXD→LDLPFC^a^ | < 0.001 | 0.002 | .832 | -0.005 | 0.004 |
| **bmTBI severity→Right dlPFC (C)** | -0.026 | 0.037 | .490 | -0.086 | 0.035 |
| *PSY-5-RF Indirect Effects* |  |  |  |  |  |
| C' | -0.017 | 0.039 | .668 | -0.094 | 0.060 |
| bmTBI severity→INTR-r→RDLPFC | -0.006 | 0.010 | .536 | -0.026 | 0.013 |
| bmTBI severity→AGGR-r→RDLPFC | -0.002 | 0.005 | .636 | -0.012 | 0.007 |
| bmTBI severity→PSYC-r→RDLPFC | 0.002 | 0.003 | .583 | -0.005 | 0.009 |
| bmTBI severity→DISC-r→RDLPFC | < 0.001 | 0.001 | .933 | -0.003 | 0.003 |
| bmTBI severity→NEGE-r→RDLPFC | -0.002 | 0.006 | .740 | -0.014 | 0.010 |
| *H-O Indirect Effects* |  |  |  |  |  |
| C'^a^ | -0.010 | 0.039 | .792 | -0.087 | 0.066 |
| C'^b^ | -0.004 | 0.041 | .912 | -0.084 | 0.075 |
| bmTBI severity→EID→RDLPFC^a^ | -0.016 | 0.009 | .094^†^ | -0.034 | 0.003 |
| bmTBI severity→RCd→RDLPFC^b^ | 0.002 | 0.014 | .882 | -0.025 | 0.029 |
| bmTBI severity→RC2→RDLPFC^b^ | -0.018 | 0.018 | .318 | -0.053 | 0.017 |
| bmTBI severity→RC7→RDLPFC^b^ | -0.006 | 0.010 | .592 | -0.026 | 0.015 |
| bmTBI severity→THD→RDLPFC^a^ | 0.001 | 0.004 | .847 | -0.008 | 0.009 |
| bmTBI severity→BXD→RDLPFC^a^ | < 0.001 | 0.002 | .828 | -0.004 | 0.003 |
| **bmTBI severity→vmPFC-sgACC (C)** | -0.044 | 0.042 | .295 | -0.114 | 0.025 |
| *PSY-5-RF Indirect Effects* |  |  |  |  |  |
| C' | -0.031 | 0.049 | .525 | -0.126 | 0.064 |
| bmTBI severity→INTR-r→VMSG | -0.006 | 0.015 | .716 | -0.035 | 0.024 |
| bmTBI severity→AGGR-r→VMSG | -0.007 | 0.009 | .413 | -0.024 | 0.010 |
| bmTBI severity→PSYC-r→VMSG | -0.002 | 0.004 | .703 | -0.009 | 0.006 |
| bmTBI severity→DISC-r→VMSG | < 0.001 | 0.005 | .933 | -0.010 | 0.009 |
| bmTBI severity→NEGE-r→VMSG | 0.001 | 0.007 | .894 | -0.013 | 0.015 |
| *H-O Indirect Effects* |  |  |  |  |  |
| C'^a^ | -0.039 | 0.045 | .382 | -0.128 | 0.049 |
| C'^b^ | -0.035 | 0.046 | .442 | -0.126 | 0.055 |
| bmTBI severity→EID→VMSG^a^ | -0.005 | 0.010 | .618 | -0.024 | 0.014 |
| bmTBI severity→RCd→VMSG^b^ | 0.002 | 0.018 | .895 | -0.034 | 0.039 |
| bmTBI severity→RC2→VMSG^b^ | -0.016 | 0.020 | .408 | -0.055 | 0.022 |
| bmTBI severity→RC7→VMSG^b^ | 0.006 | 0.012 | .624 | -0.017 | 0.028 |
| bmTBI severity→THD→VMSG^a^ | < 0.001 | 0.001 | .926 | -0.002 | 0.002 |
| bmTBI severity→BXD→VMSG^a^ | < 0.001 | 0.001 | .930 | -0.003 | 0.002 |

Note: Predictors were z-scored prior to inclusion in the model. Full set of multiple mediator estimates shown for significantly moderated paths from Model 2 only. ^a^Indirect-only path. ^b^Estimates from Model 3a. ^c^Estimates from Model 3b. P-values shown here are uncorrected. ^†^*p* < .10. **p* < .05. ***p* < .01. ****p* < .001.

Table S14. *Model 3b* *standardized inter-region correlations*

|  | Left Amygdala | Right Amygdala | Left  dlPFC | Right dlPFC | vmPFC-sgACC |
| --- | --- | --- | --- | --- | --- |
| Left Amygdala | -- |  |  |  |  |
| Right Amygdala | **0.277***** | -- |  |  |  |
| Left dlPFC | **0.046***** | **0.074***** | -- |  |  |
| Right dlPFC | 0.007 | 0.023 | **0.058***** | -- |  |
| vmPFC-sgACC | **0.141***** | **0.166***** | **0.053***** | 0.006 | -- |

Note: P-values shown here are uncorrected. ^†^*p* < .10. **p* < .05. ***p* < .01. ****p* < .001.

Table S15. *Statistical details of inter-region correlations in Model 3b*

|  |  |  |  | 95% CI | |
| --- | --- | --- | --- | --- | --- |
|  | β | SE(β) | *p* | Low | High |
| **Left Amygdala 🡨🡪 Right Amygdala** | **0.277** | **0.033** | **< .001***** | **0.223** | **0.330** |
| **Left dlPFC 🡨🡪 Right dlPFC** | **0.058** | **0.010** | **< .001***** | **0.039** | **0.077** |
| **Left Amygdala 🡨🡪 Left dlPFC** | **0.046** | **0.011** | **< .001***** | **0.029** | **0.064** |
| Left Amygdala 🡨🡪 Right dlPFC | 0.007 | 0.014 | .603 | -0.016 | 0.030 |
| **Right Amygdala 🡨🡪 Left dlPFC** | **0.074** | **0.015** | **< .001***** | **0.050** | **0.099** |
| Right Amygdala 🡨🡪 Right dlPFC | 0.023 | 0.017 | .176 | -0.005 | 0.051 |
| **vmPFC-sgACC 🡨🡪 Left Amygdala** | **0.141** | **0.030** | **< .001***** | **0.092** | **0.190** |
| **vmPFC-sgACC 🡨🡪 Right Amygdala** | **0.166** | **0.032** | **< .001***** | **0.113** | **0.218** |
| **vmPFC-sgACC 🡨🡪 Left dlPFC** | **0.053** | **0.011** | **< .001***** | **0.034** | **0.071** |
| vmPFC-sgACC 🡨🡪 Right dlPFC | 0.006 | 0.014 | .673 | -0.017 | 0.028 |

Note: P-values shown here are uncorrected. ^†^*p* < .10. **p* < .05. ***p* < .01. ****p* < .001.

Table S16. *Conceptual families of research questions subjected to Bonferroni correction in Model 3b*

| Effect Type | Condition | Research Question Prefix | Tests |
| --- | --- | --- | --- |
| A path (Direct Effects) | 0-back Neutral | What is the direct effect of THD (Thought Dysfunction) on… | 1. Left amygdala? 2. Right amygdala? 3. Left dlPFC? 4. Right dlPFC? 5. VmPFC-sgACC? |
|  | 2-back Neutral | What is the direct effect of RCd (Demoralization) on… | 1. Left amygdala? 2. Right amygdala? 3. Left dlPFC? 4. Right dlPFC? 5. VmPFC-sgACC? |
|  |  | What is the direct effect of RC2 (Low Positive Emotions) on… | 1. Left amygdala? 2. Right amygdala? 3. Left dlPFC? 4. Right dlPFC? 5. VmPFC-sgACC? |
|  | 0-back Combat | What is the direct effect of RC7 (Dysfunctional Negative Emotions) on… | 1. Left amygdala? 2. Right amygdala? 3. Left dlPFC? 4. Right dlPFC? 5. VmPFC-sgACC? |
| C’ path (Partial Direct Effects) | 0-back Neutral | After including the H-O scales, does PTSS continue moderating the activity of… | 1. Left amygdala? 2. Right amygdala? 3. Left dlPFC? 4. Right dlPFC? 5. VmPFC-sgACC? |
|  | 2-back Neutral | After including the H-O scales, does PTSS continue moderating the activity of… | 1. Left amygdala? 2. Right amygdala? 3. Left dlPFC? 4. Right dlPFC? 5. VmPFC-sgACC? |
|  | 0-back Combat | After including the H-O scales, does bmTBI continue moderating the activity of… | 1. Left amygdala? 2. Right amygdala? 3. Left dlPFC? 4. Right dlPFC? 5. VmPFC-sgACC? |
| A*B path (Indirect Effect) | 0-back Neutral | Are PTSS-related decreases in left amygdala attributable to… | 1. RCd? 2. RC2? 3. RC7? 4. THD? 5. BXD? |
|  |  | Are PTSS-related decreases in right amygdala attributable to… | 1. RCd? 2. RC2? 3. RC7? 4. THD? 5. BXD? |
|  |  | Are PTSS-related decreases in vmPFC-sgACC attributable to… | 1. RCd? 2. RC2? 3. RC7? 4. THD? 5. BXD? |
|  | 2-back Neutral | Are PTSS-related increases in left amygdala responses attributable to… | 1. RCd? 2. RC2? 3. RC7? 4. THD? 5. BXD? |
|  |  | Are PTSS-related increases in right amygdala responses attributable to… | 1. RCd? 2. RC2? 3. RC7? 4. THD? 5. BXD? |
|  |  | Are PTSS-related increases in left dlPFC responses attributable to… | 1. RCd? 2. RC2? 3. RC7? 4. THD? 5. BXD? |
|  |  | Are PTSS-related increases in right dlPFC responses attributable to… | 1. RCd? 2. RC2? 3. RC7? 4. THD? 5. BXD? |
|  | 0-back Combat | Are bmTBI-related decreases in left amygdalar responses attributable to… | 1. RCd? 2. RC2? 3. RC7? 4. THD? 5. BXD? |
|  |  | Are bmTBI-related decreases in right amygdalar responses attributable to… | 1. RCd? 2. RC2? 3. RC7? 4. THD? 5. BXD? |

*.*

Figure S1.


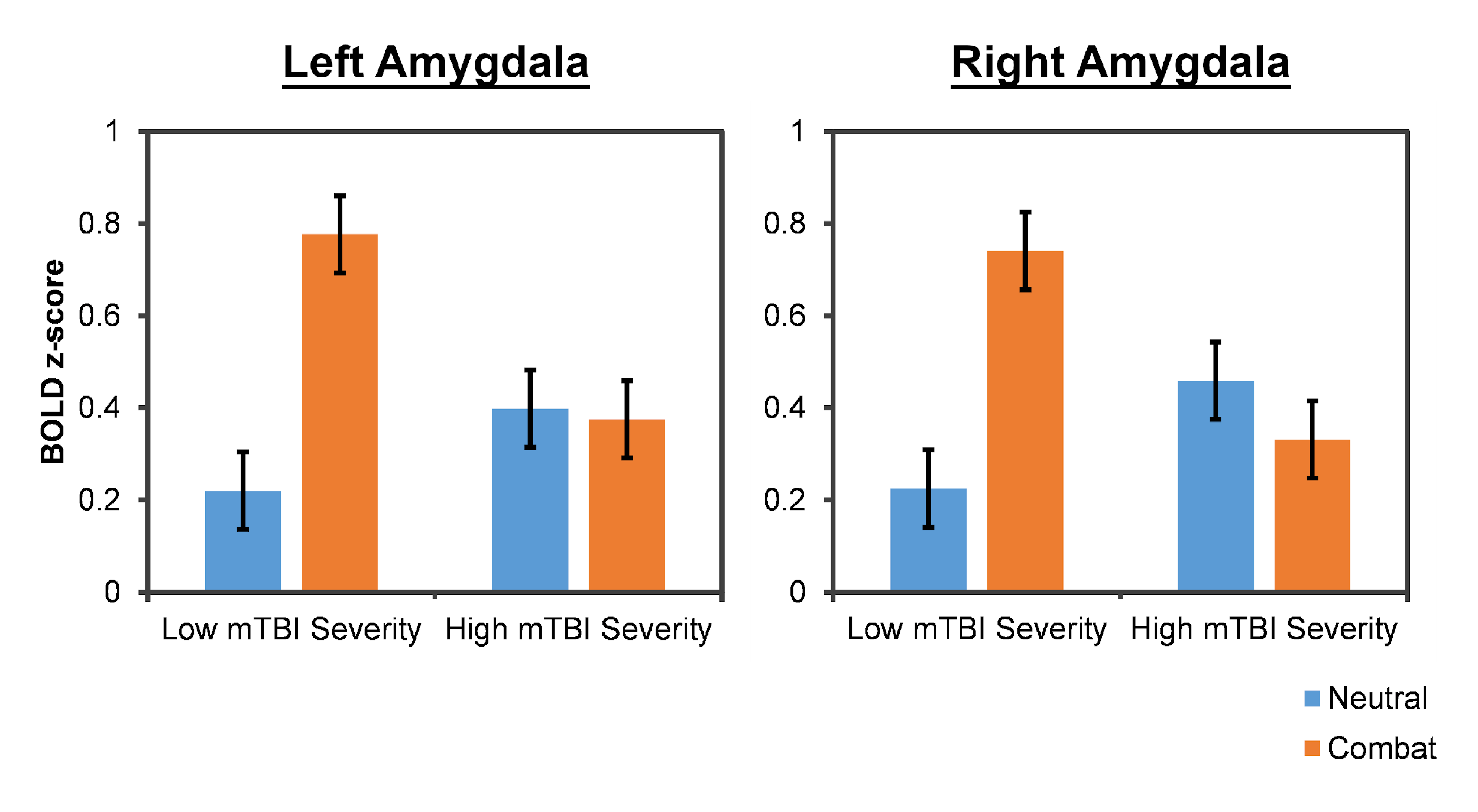


Note: Means and standard errors are displayed. Increases in bmTBI severity scores were associated with less activation in the left amygdala in the Affect (0-back-combat images) condition. Right amygdalar activity exhibited the same pattern as a function of bmTBI severity scores. BOLD = blood-oxygen-level-dependent, mTBI = mild traumatic brain injury.

Figure S2.


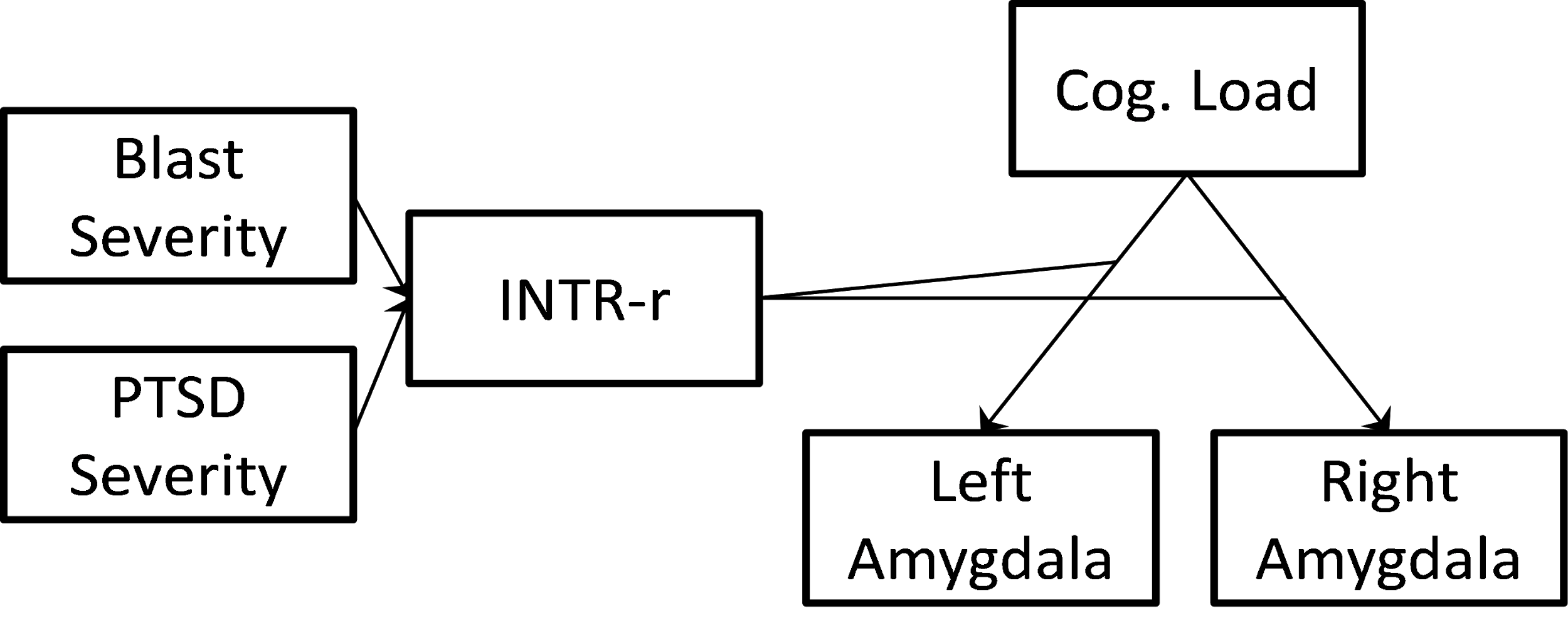


Note: Simplified path diagram for model S3. Only significant paths to study effects via PSY-5-RF scales are shown. INTR-r partially mediates the CAPS Severity-associated increases in bilateral amygdala under cognitive load. Cog. = 2-back neutral image condition predictor, PTSD = posttraumatic stress disorder, INTR-r = Introversion.

Supplemental References

Tamm, L., Narad, M. E., Antonini, T. N., O’Brien, K. M., Hawk, L. W., & Epstein, J. N. (2012). Reaction time variability in ADHD: A review. *Neurotherapeutics*, *9*(3), 500–508.
